# System dynamic modelling of healthcare associated influenza -a tool for infection control

**DOI:** 10.1186/s12913-022-07959-7

**Published:** 2022-05-27

**Authors:** Martina Sansone, Paul Holmstrom, Stefan Hallberg, Rickard Nordén, Lars-Magnus Andersson, Johan Westin

**Affiliations:** 1 Department of Infectious Diseases, Institute of Biomedicine, Sahlgrenska Academy, University of Gothenburg, Guldhedsgatan 10B, 413 46 Gothenburg, Sweden; 2 Department of Infectious Diseases, Region Vastra Gotaland, Sahlgrenska University Hospital, Journalvagen 10, 416 50 Gothenburg, Sweden; 3 Department of Radiation Physics, Institute of Clinical Sciences, Sahlgrenska Academy, Gothenburg University Medicinaregatan 3, 413 45 Gothenburg, Sweden; 4 Regional Cancer Centre West, Western Sweden Healthcare Region, 413 45 Gothenburg, Sweden; 5 Department of Clinical Microbiology, Region Vastra Gotaland, Sahlgrenska University Hospital, Guldhedsgatan 10A, 402 34 Gothenburg, Sweden

**Keywords:** Healthcare-associated infections, Influenza, Infection prevention and control, System dynamics, Decision support systems, Modelling

## Abstract

**Background:**

The transmission dynamics of influenza virus within healthcare settings are not fully understood. Capturing the interplay between host, viral and environmental factors is difficult using conventional research methods. Instead, system dynamic modelling may be used to illustrate the complex scenarios including non-linear relationships and multiple interactions which occur within hospitals during a seasonal influenza epidemic. We developed such a model intended as a support for health-care providers in identifying potentially effective control strategies to prevent influenza transmission.

**Methods:**

By using computer simulation software, we constructed a system dynamic model to illustrate transmission dynamics within a large acute-care hospital. We used local real-world clinical and epidemiological data collected during the season 2016/17, as well as data from the national surveillance programs and relevant publications to form the basic structure of the model. Multiple stepwise simulations were performed to identify the relative effectiveness of various control strategies and to produce estimates of the accumulated number of healthcare-associated influenza cases per season.

**Results:**

Scenarios regarding the number of patients exposed for influenza virus by shared room and the extent of antiviral prophylaxis and treatment were investigated in relation to estimations of influenza vaccine coverage, vaccine effectiveness and inflow of patients with influenza. In total, 680 simulations were performed, of which each one resulted in an estimated number per season. The most effective preventive measure identified by our model was administration of antiviral prophylaxis to exposed patients followed by reducing the number of patients receiving care in shared rooms.

**Conclusions:**

This study presents an system dynamic model that can be used to capture the complex dynamics of in-hospital transmission of viral infections and identify potentially effective interventions to prevent healthcare-associated influenza infections. Our simulations identified antiviral prophylaxis as the most effective way to control in-hospital influenza transmission.

**Supplementary Information:**

The online version contains supplementary material available at 10.1186/s12913-022-07959-7.

## Background

The annual global influenza epidemic has a great impact on the society in terms of increased morbidity, mortality and cost. For healthcare facilities, influenza infections pose special hazards as acute-care hospitals are semi-closed, crowded settings with a continuous internal flow of people. Transmission of infectious diseases is a complex interplay determined by the infectivity of the pathogen, the contagiousness of the infected individual, the susceptibility of the exposed individual, the contact patterns between the infected individual and the exposed individual and the environmental stress exerted on the pathogen during transmission [1]. These factors may act in conjunction to develop “super-spreading events”; where a few individuals disproportionally infect several secondary cases [2]. Reduced transmission therefore by extension also reduces influenza-related morbidity and mortality rates. Healthcare-associated infections (HCAI), sometimes referred to as nosocomial or hospital-acquired infections, are a threat to both patients and caregivers, and account for an annual estimated number of 330,000 deaths and 900,000 disability-adjusted life years in Europe only [3]. Hospital in-patients often have underlying illnesses which make influenza infections more severe and potentially fatal in this setting [4, 5], and in addition to patients may staff and visitors act as potential reservoirs. The current COVID-19 pandemic has put focus on environmental factors such as the extent of airborne transmission, which has been a long-standing controversy regarding influenza virus [6, 7]. Healthcare-associated infections caused by influenza viruses is likely to be underrecognized [8, 9] and there are considerable differences regarding case definitions, surveillance methods and infection control practices [9] which may complicate development of evidence-based policies.

To control in-hospital transmission and prevent outbreaks, the possibility of forecasting plausible scenarios is crucial for healthcare planning. Modelling studies may be used to illustrate patterns, facilitate understanding and assist decision-making [10]. Furthermore, modelling studies have the advantage of being cost-effective and ethically feasible as they do not put patients or staff at risk. Statistical and mathematical models however have limitations regarding the non-linear connections and multiple interactions that characterizes complex real-world situations like infectious disease transmission. Instead, system dynamic modelling may be used, which by generating quantitative results using interacting scales and feedback loops within defined boundaries [11, 12]. Computational tools are essential to synthesize data for modelling. Several software applications are now available, which may include free on-line publication of model interfaces. System dynamic modelling has been used in different settings for refining guidelines and designing prevention strategies [13–16]. It has been described as well suited for medical research due to the complex and feedback-rich interactions and enables compression of decade-long disease trajectories into very short time, testing sensitivities and combinations of interventions [17, 18]. Simulation models for transmission of HCAI have previously focused on bacterial infections, depicting single-ward settings such as intensive-care units [17, 19–22]. Regarding healthcare-associated influenza, only a few modelling studies have been found [23–26], to our knowledge none with focus on transmission between patients.

The aim of this study was to develop an applicable system dynamic model to illustrate the in-hospital transmission pattern of influenza across an entire season. Moreover, we aimed to use the model to simulate various scenarios in order to predict the relative impact of modifiable factors and to identify effective measures for preventing transmission of healthcare-associated influenza.

## Methods

The basic structure of the model described in this paper was constructed in collaboration between the authors, which include clinical expertise in infectious diseases, virology, infection control and experienced system dynamic modellers. It was designed exclusively for this study and integrates virologic properties and national surveillance data. The Stella Architect simulation software (Stella Architect, version 1.7.1, isee systems Inc., Lebanon, NH, USA) was utilized to produce estimates of the total number of healthcare-associated influenza cases during a typical season for a variety of possible scenarios.

The process consisted of the following consecutive steps:

1. Methodological considerations. 2. Identifying key variables with a potential influence on in-hospital transmission of influenza 3. Construction and technical validation of the model. 4. Selecting the model scenarios of interest. 5. Producing the simulations.

### Methodological considerations

Initially three simulation methodologies were considered. Firstly, discrete event simulation, which may be used to model sequences of activities and specific events over time [17] Discrete event simulation represents systems at an operational level [27] with stochastic changes in discrete intervals [28] which was not the case in the project at hand. Secondly, agent-based modelling, where agents (patients) are described by their properties and interactions with other agents [17] . Agent based models may be useful when simulating epidemiology [29] although resource-intensive in both the modelling and interpretation phases [30]. Finally, a system dynamic model was selected as it may illustrate continuous flows rather than individual events [27], aggregated data and non-linear relationships [17] which are characteristic for transmission of infectious diseases. Furthermore, the authors had both solid experience of system dynamic modelling of disease trajectories [15] as well as access to data for healthcare-associated influenza for season 2016/17 [31].

### Key variables used for model design

The Sahlgrenska University Hospital, Gothenburg, Sweden constitutes the base of the model regarding patient flow and clinical management. It is a full-scale, acute-care facility with ~ 1900 beds, three separate emergency departments (ED) and has daily access to diagnostic virology laboratory service. The internal medicine ward housing the highest number of influenza-infected patients during previous seasons served as a model for a standard ward regarding data for mean bed occupancy rate, facility design (number of patients in single occupancy rooms vs rooms with multiple beds) and a mean length-of-hospital stay of 3 days. All patients at the standard ward are admitted via the ED. During the peak influenza season (December–March), the standard ward had an average occupancy rate of 110%, a total of 1031 hospitalizations and 55 cases of confirmed influenza. Due to the high occupancy rate, cohorting of patients with influenza in multiple rooms is common instead of single room care.

Data regarding patient flow from season 2016/17 was collected from the hospital administrative system. The average number of patients seeking care at the main ED were estimated to be 4600 per month, whereof 26% were admitted for inpatient care. Number of patients with symptoms possibly explained by influenza (fever, shortness of breath or “unspecified infection” as registered reason for encounter) were estimated to be 600 cases per month. We assumed that the average time point for ED consultation occurred at day 2 of the disease course for an influenza patient, which is in line with other reports [32–34]. Exposure was defined as contact by sharing room with a confirmed influenza as suggested by the hospital guidelines for influenza prevention and control. The average number of roommates for each patient at the standard ward was 2.2, and we used this figure as a proxy of the number of exposed patients per influenza case.

The definition of an influenza case was any patient > 18 years old with a respiratory sample positive for influenza by real-time PCR. A case of HCAI was (in addition to a laboratory confirmed infection) was defined as having onset of influenza-like or acute respiratory symptoms > 48 h after hospital admission or < 48 h after a previous discharge, according to current definitions proposed by US Centers for Disease Control and Prevention [35]. We assumed that individual infectivity was related to the graph showing the change in viral load in nasopharynx samples during the disease course, why we used this as proxy measure [36, 37].

The mean effect of antiviral treatment of symptomatic infection was estimated to shorten the duration of symptoms (which in the model was assumed to follow the curve for viral load interpreted as infectivity) by 24 h when treated within 48 h after symptom onset [38]. A share of uninfected individuals may further develop influenza despite antiviral prophylaxis. Recommended prophylactic treatment after exposure (oseltamivir) is estimated to reduce the risk of infection for exposed individuals by 70–90% [39], why we chose a mean of 80% effect as model variable. Epidemic curves showing weekly estimates of the number of confirmed influenza cases in the society over time from season 2013–2019 were collected from the Public Health Agency of Sweden [40].

Finally, we integrated previously published real-world outcome data for management of influenza cases at the hospital season during the 2016/17 season [31]. This particular season, a total of 432 hospitalized cases of influenza were identified at the hospital, whereof 114 (26%) were classified as HCAI. Of the non-HCAI influenza cases, 53% were treated with antiviral therapy compared with 62% of the HCAI-cases. Based on the recommendation to offer treatment to all patients in need of hospital care for influenza, we therefore estimated the diagnostic accuracy at the ED to be 56% based on clinical presentation and management.

The risk to be infected with influenza during hospital stay for patients who were not infected at admission further depend on vaccination coverage and vaccine effectivity, which vary between seasons. We therefore included the mean vaccine effectiveness for the season 2016/17, which was estimated to be 40% [41], and national data regarding vaccine coverage among people > 65 years old which were estimated to be 49% [40].

Several unknown factors are likely to influence the number of HCAI cases during an influenza season. Aerosol transmission may occur over longer distances and has been suggested to account for approximately half of the transmission events [42], but is highly depending on local environmental conditions. In addition, the potential for health-care workers to act as reservoirs or vectors for transmission of influenza is high [43] but insufficiently studied.

### Construction and technical validation

**Fig. 1:**
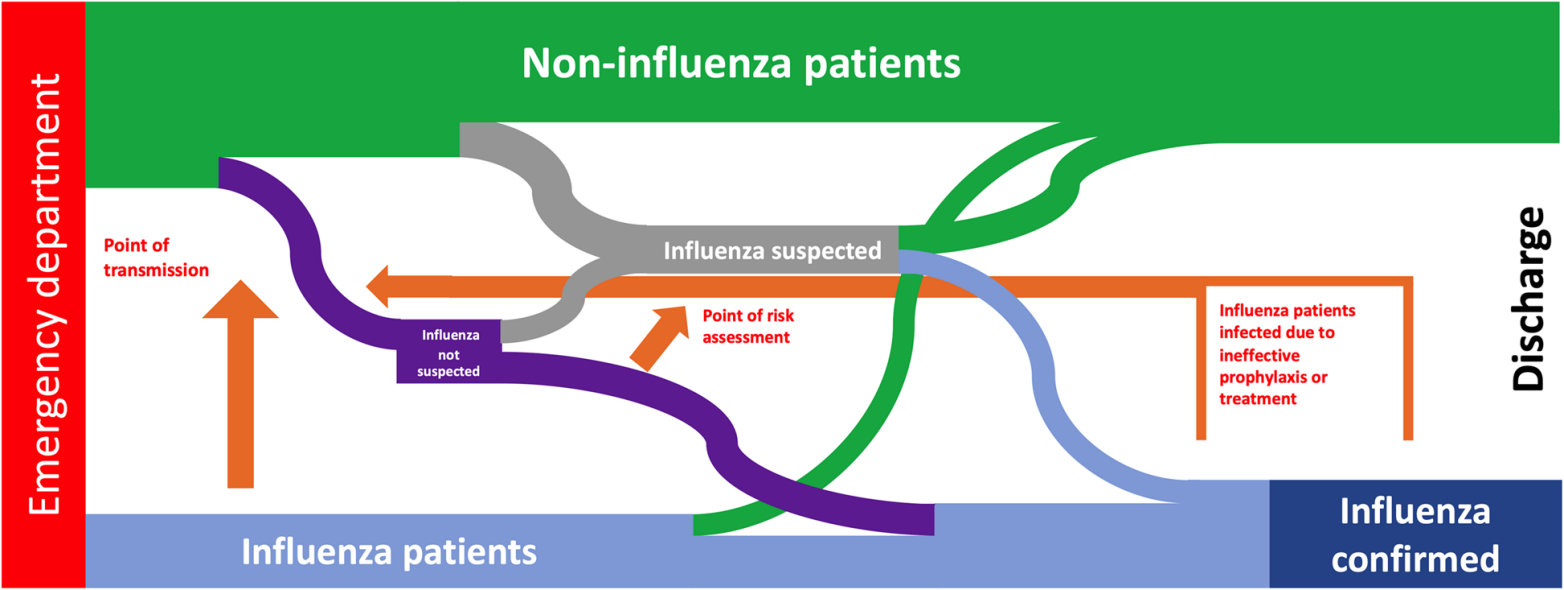
Flow-chart of the patient populations. Patients not infected with influenza on admission to the hospital are shown in green. Patients with influenza (blue) may be either detected or undetected. Influenza may be transmitted from the infected patients to non-infected, mainly by close contact by sharing rooms (purple). Patients with a known exposure to influenza (grey) are recommended prophylactic antiviral treatment. Some of the exposed patients yet develop influenza and thereby transfer to the blue flow, others remain in the green flow. A small number of influenza patients may recover during hospitalization (transferring from the blue flow to the green), others remain in the blue flow and are discharged while still infectious

**Fig. 2:**
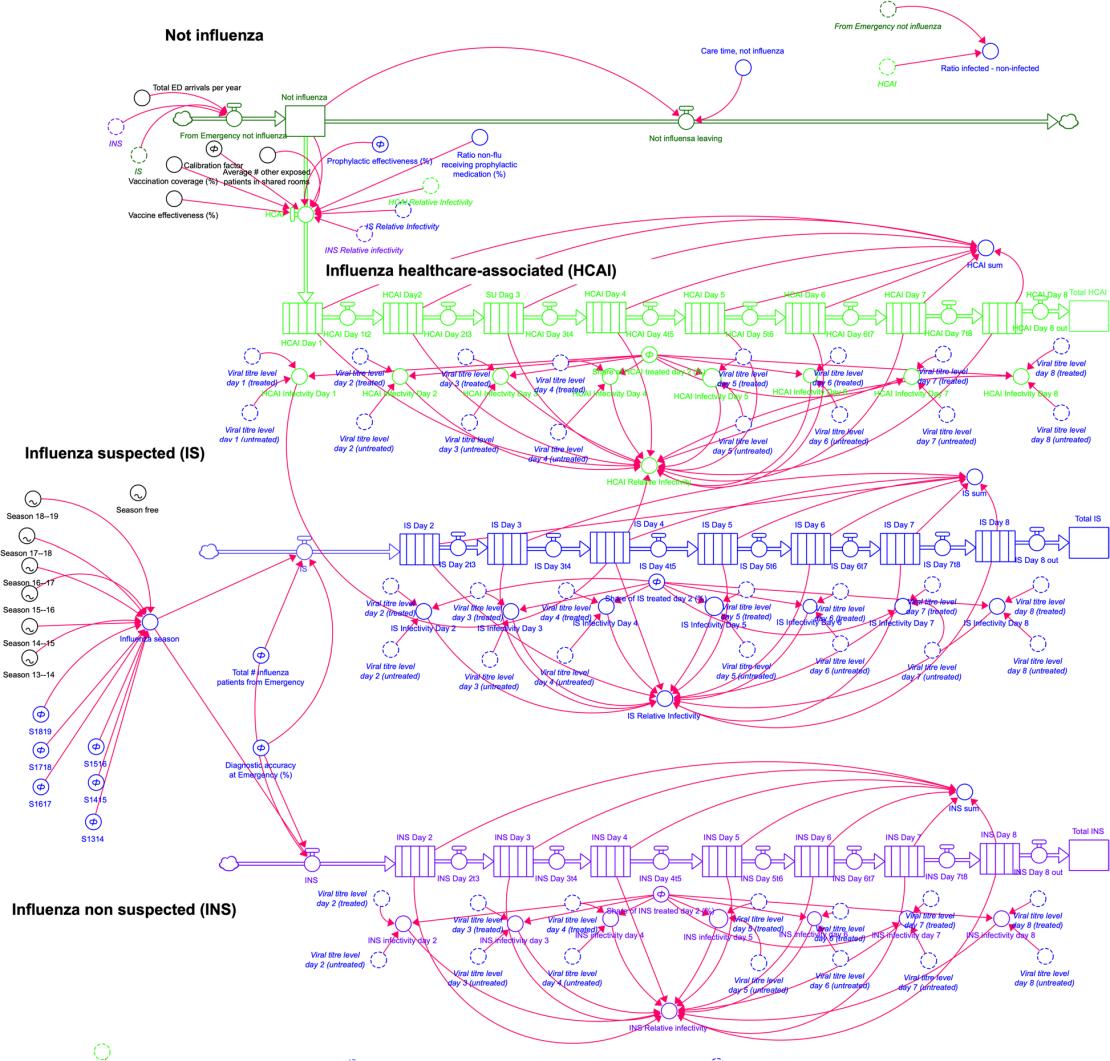
“Stock-and flow” model. Stocks (accumulations of patients) are illustrated as rectangles, and flows (inputs and outputs) as pipes to/from the stock as the stages change. The model structure follows Fig. 1. The inflow of influenza patients is divided into two sub-flows, (1) patients where influenza is suspected already at the ED and may thereby be subject to immediate interventions such as antiviral treatment and/or single room care and (2) patients who are not suspected of having influenza. Each flow has an “aging chain” over 7 days, (where infectivity is reflected by a curve for viral load day-by-day). Patients admitted for other reasons may be exposed by sharing rooms with infected patients. Individual infectivity is included as a function of the average viral loads of infected patients, vaccination effectivity, coverage and effectiveness of antiviral prophylactic treatment. Patients who become infected at the hospital also flow into an “aging chain” where they subsequently may infect other fellow patients

The construction of the model started with illustrating the patients’ way from the ED through the hospital until discharge by a simple flow chart (Fig. 1), which the modellers then translated into a “stock-and-flow” diagram (Fig. 2.) The purpose was to highlight when and how influenza may spread to other patients within the hospital and builds upon a graphical representation which illustrates the patient-flows involved. Details of exact time-point for transmission, or when suspicion for influenza arises, differs among patients and is often unknown. Figure 1 was used primarily to establish a common ground and ensure that the modellers understood the patient flows and the interventions which should be reflected in the finalized system dynamic model.

The “stock-and-flow”- diagram is more extensive and less easy to comprehend without specific knowledge, but enables quantifications of scenarios by mathematical expressions and interactions where both actual data and assumptions can be combined. The different patient populations constitute the major flows in the system. Stocks represent groups of patients at a similar stage (in this model divided into cohorts day-by-day along the disease course), what in system dynamics is described as an “aging chain”. The model has in total 32 flows connecting 33 stocks mainly along aging chains. The stocks and 54 variables are used in 40 equations influencing the hitherto non-infected patients. A key factor in the model was the actual “attack rate”, which was handled through by adding a calibration factor to match the true number of healthcare-associated infections found during season 2016/17. Thus, in this respect, the “calibration factor” reflect several random accumulated unknown variables with a potential impact on in-hospital influenza transmission.

The validity of a model constructed for evaluating effectiveness of strategies refers primarily the internal structure, which should represent the relevant aspects as well as reproducing and explaining the behaviour of the system [44]. The structural validity of the model was ensured by ascertaining its congruence with the patient flows in Fig. 1. The behavioural validity was established by thorough testing by the medical researchers ensuring that the model behaved as expected as well as testing extreme values in input variables. During validation the model went through four major iterations and numerous minor iterations. In our model, the non-infected patient population is infected by individuals included in an influenza-infected population. Individual infectivity (reflected by the curve for viral load day-by-day) were included in the model by the aging chains. The resulting number of HCAI cases further depend on exposure.

Apart from studying the effects of the variables mentioned above, we included a possibility to set various seasonal epidemic curves in the model as well as increasing the total number of influenza-infected patients seeking care at the ED. Rather than adding additional randomness we decided to include the total number of influenza cases from previous seasons as this would allow us to future studying of seasons as single scenarios. By this mean the model may compare if interventions provide similar outcomes despite seasonal differences. Furthermore, the user interface allows testing the effects of an alteration of any variable, either isolated or in combination with alterations of other variables. Equations, an expanded version of Fig. 2 including model interface and an xmile version of the model are included in the Additional files 1 and 2.

### Selection of the model scenarios of interest

#### **Step 1:** Impact of modifiable patient-related factors

Our purpose was to construct a model able to identify the most effective control measures *for a hospital* to reduce the total number of HCAI cases per season. We therefore initially focused on modifiable patient-related factors and stepwise altered following four scenarios in the model: (i) Mean number of patients exposed by sharing room with an influenza case (ii) Share of non-HCAI cases receiving antiviral treatment within 48 h of symptom onset (iii) Share of HCAI influenza cases receiving antiviral treatment within 48 h of symptom onset (iv) Share of exposed patients receiving antiviral prophylaxis.

#### **Step 2:** Impact of non-modifiable epidemiological factors

In the second simulation round, the variables identified as having the most impact in simulation one, i.e., mean number of exposed patients per influenza case and share of exposed subjects receiving antiviral were retained. We instead added and stepwise altered extreme values of scenarios beyond hospital control (non-modifiable) for mean vaccine coverage, mean vaccine effectiveness and total number of influenza cases seeking care at the ED.

### Producing the simulations

One variable at a time were given a set value (for example: mean number of exposed patients = 1), followed by a stepwise altering of the other scenarios selected above, one variable at a time. Each simulation was performed by manually changing the variable on the screen which instantly delivered instant numerical and graphical results as total number of HCAI cases per season. This leaves 13–15 estimates per set variable. In the first round, a total of 240 simulations were performed followed by 440 in the second round.

## Results of simulations

Basic model variables are shown in Table 1A-C and results of the simulations are summarized in Table 2. The outcome variable is presented as the estimated total number of HCAI cases per season. Antiviral prophylaxis given to patients who were exposed by sharing room with an influenza case was identified as the single most effective measure, followed by a reduction of the mean number of exposed patients. Table 2 summarizes the estimated number of HCAI cases for the simulated scenarios by stepwise alteration of these two variables. If antiviral prophylaxis was administered to all exposed patients, approximately 17 cases of HCAI would occur. This is explained by a mean number of one (not zero) exposed patients/influenza case combined with known incomplete protective effect of prophylaxis [45]. The maximum number of estimated HCAI-cases in simulation round 1 was found to be 432 in a scenario where no exposed patients received prophylaxis and three patients were exposed per influenza case. Antiviral treatment of already asymptomatic non-HCAI, as well as of HCAI-cases, had limited effect on in-hospital transmission in the model (data not shown).

**Table 1:** **A.** Basic model variables based upon seasonal data from 2016/17. **B**. Altered variables in simulation round 1. **C**. Altered variables in simulation round 2

**A. Basic model variables**	
Influenza cases (n)	432
Mean number exposed in shared rooms (n)	2.2
Vaccine coverage (%)	49
Vaccine effectiveness (%)	40
Share of exposed treated with prophylaxis < 48 h (%)	56
Prophylactic effectivity (%)	80
Diagnostic accuracy at ED (%)	56
Share of non-HCAI influenza treated on admission (%)	53
Share of HCAI influenza treated < 48 h (%)	62
**B. Simulation round 1**	
(1) Mean number exposed in shared rooms (n)	1–2- 3
(2) Share of non-HCAI treated on admission (%)	0–25–50-75-100
(3) Share of HCAI treated < 48 h (%)	0–25–50-75-100
(4) Share of exposed receiving prophylaxis (%)	0–25–50-75-100
**C. Simulation round 2**	
(1) Mean number exposed in shared rooms (n)	1–2- 3
(2) Share of exposed receiving prophylaxis (%)	0–25–50-75-100
(3) Mean vaccine coverage (%)	0–25–50-75-100
(4) Mean vaccine effectiveness (%)	0–25–50-75-100
(5) Total influenza inflow to ED (n)	500–1000–1500-2000

**Table 2:** Estimated number of HCAI influenza cases found by modelling scenarios by altering mean number of exposed patients in shared rooms in relation to share of exposed patients receiving antiviral prophylaxis

	100%	75%	50%	25%	0%
**Mean exposed patients/room (n)**					
1	**17**	34	53	74	92
2	35	75	121	134	235
3	54	121	203	304	**432**

The differences in total number of healthcare-associated influenza cases per simulated season when the extent of antiviral prophylaxis was set to 0–25–50-75-100% for the selected variables in simulation round 1 were 415 (range 17–432) and 522 (range 17–539) in simulation round 2. The substantial impact of the extent of antiviral prophylaxis initiated after exposure found in our model was also well demonstrated by an estimated number of HCAI of less than 100 despite a worst-case model scenario; including variables set to 0% vaccine coverage, 0% vaccine effectiveness, a mean number of 3 exposed cases/ influenza case and a total inflow of 2000 patients with influenza symptoms to the hospital ED.

We further estimated the risk for patients admitted for other reasons of contracting influenza during hospital-stay and applied this for different model scenarios. Based on the hospital data from 2016 to 17, the following calculations were made. The influenza season was assumed to last for 12 weeks. The total number of patients admitted during this season was estimated to be 3588 (on average 4600/month ED appointments with an admittance rate of 26%). The number of non-HCAI influenza cases were found to be 321, which leaves a total of 3588–321 = 3267 patients at risk of acquiring influenza during hospital stay. The number of HCAI influenza cases where 114, which leaves an estimated risk of 3.5% for any patient during the influenza season, not infected upon admittance, to develop influenza during hospitalization. By increasing the share of prophylaxis from 0 to 100%, the risk for contracting influenza decreased as followed (all other variables left unchanged): Mean number of exposed cases: One: 2.8–0.5% Two: 7.2–1.1% and Three: 13.2–1.7%. We selected two future scenarios for risk calculations: Mean number (1, 2 and 3) of exposed patients in shared rooms in relation to share of exposed patients receiving antiviral prophylaxis (0–100%). In Table 3, the model estimates for absolute and relative risk reductions are displayed in addition to relative risk and number of patients needed to treat to prevent one HCAI influenza case.

**Table 3:** Risk reduction and NNT for contracting influenza during hospital-stay. Numbers shown for HCAI influenza cases in relation to mean number of exposed cases (1, 2 and 3) and effect of increased share of exposed cases receiving prophylaxis (0–100%)

Mean exposed (n)	HCAI (n) Prophylaxis 0%	HCAI (n) Prophylaxis 100%	ARR	RRR	RR	NNT
**1**	92	17	0.02	0.81	0.19	45
**2**	235	33	0.06	0.85	0.15	18
**3**	432	54	0.10	0.86	0.14	10

*ARR* Absolute risk reduction, *RRR* Relative risk reduction, *RR* Relative risk and *NNT* Number needed to treat

## Discussion

In this study, we have constructed a system dynamic model for healthcare-associated influenza. We show how the model may be used to increase the understanding of in-hospital influenza transmission, make predictions of future scenarios, and estimate the effect of preventive interventions in a typical hospital.

We chose to construct a system dynamic model, due the ability to capture the non-linear relationships and feed-back loops [11] which applies well for transmission of infectious diseases. It was the first type of simulation model published to illustrate healthcare associated infections and one of the most widely used [46]. Alternative modelling strategies were discussed but considered less suitable for the case at hand. A future approach may instead be models which combine discrete events for patient’s logistics with system dynamics for epidemiology suggested by Viana et al. [47].

Previous evaluations of healthcare-associated influenza are often focused on single components in the chain of transmission and have found moderate to low effect of control measures such as hand hygiene, facemasks, and vaccination of health-care workers [48–50]. Observational studies of influenza transmission and hospital outbreaks often have shortcomings in methodology and multiple confounders. Communicable diseases differ from non-communicable diseases in the aspect of interventions which reduces the number of cases in one population (such as direct effects of vaccination or prophylaxis) will further reduce the risk of other in-patients becoming infected (indirect effects). Dynamic models may reproduce this complexity of on transmission and was therefore preferred over a static model which assume a constant force of infection.

The finding of antiviral prophylaxis as an effective measure to reduce the number of HCAI cases in our model is in line with previous reports [39, 51, 52] and has been shown to shorten the duration of influenza outbreaks at long-time care facilities [53, 54]. In addition, hospitalization in double-occupancy rooms vs single-occupancy rooms showed higher risk of hospital-acquired influenza in a prospective cohort study [55]. Alterations in prophylaxis regimens may however be more easily enforced than single room care or cohorting of infected patients, especially during the seasonal peak of influenza. Likewise, high-quality evidence supporting that antiviral treatment to already symptomatic influenza patients interrupt further transmission is lacking [56].

Exposure was included as a variable in the model only for patients sharing room with an influenza case, although there may be several other opportunities for influenza exposure within the hospital environment. However, this is a common definition used by hospital infection control in non-outbreak situations. We assumed a direct association between infectivity and nasopharyngeal viral load which has been proposed in other studies [36, 57], although evidence for this is limited and might over-estimate the extent of transmission occurring around the time of symptom onset [37].

The precision in recognizing influenza cases, both at the ED as well as later during hospital stay, is a key factor to target patients who are likely to spread the virus and enable any control measure. Early identification may be even more important than prophylaxis when it comes to prioritizing single room care (or cohorting of influenza patients) in order to limit the duration of exposure. There is no established conclusion regarding the effect of oseltamivir treatment regarding on-ward transmission from patients already affected by influenza [39]. This is in line with the relatively low impact of antiviral treatment detected by our model and further underlines the importance of antiviral prophylaxis. A modelling study of hospital influenza by Blanco et al. 2016 found antiviral treatment to be less effective than hand-washing, vaccination and patient isolation, however they had not investigated antiviral treatment and prophylaxis separately [25].

All models use simplifying assumptions but need to depict real-world conditions as closely as possible to be reliable. The consistency in results made by a high number of simulations may be used as means of sensitivity. Perfect predictions of future outcomes are impossible to achieve, why the results should be interpreted more as trends although definite numbers are provided. Data for flu infectivity, vaccine effectivity, coverage, etc. are not known beforehand and may be unreliable even post-season. Other variables remain more stable across seasons, such as incubation time and treatment/prophylactic effect. True verification and validation of model fitness can however only be done retrospectively, when the actual outcome (total number of healthcare-associated influenza cases) is known. This is enabled in our model by integrating local hospital data, national surveillance data, and by the possibility to include new scenarios and modify any variable as soon as new information become available. Including seasonal updates of true HCAI numbers from our hospital may in the future be used for validation and continuous improvement of the model. To further develop the model, we also aim to introduce multivariable testing of extreme scenarios. By this mean, pandemic situations might be illustrated.

It is important to bear in mind that the aim for our model is to specifically illustrate hospital transmission of influenza with focus on patient safety, not efficiency in terms of cost-benefit. However, individual risks and benefits may apply for patients, even if they do not affect onward transmission. In analogy, prophylaxis may not be suitable for all patients. Moreover, increased use of antivirals may have ecological effects as it degrades poorly in conventional sewage processes which can contribute to antiviral resistance [58, 59]. The role of HCW’s and visitors for hospital transmission of influenza remain unclear. No data for influenza in HCW’s were available for the present study and therefore not possible to include as a variable in our model. Although numerous alternative simulation strategies were possible, the variables were selected to primarily capture hospital measures. It was assumed that testing extreme scenarios in step 2 would be more appropriate in assessing model fitness rather than the less prominent changes in seasonal curves. The simulations were carried out by testing single variables, one at a time. To adequately predict future scenarios, multiple variable testing is needed as changes in patient flow, number of exposed patients, vaccine effectivity et cetera might develop simultaneously.

System dynamic modelling is increasingly being used but it is unclear to what extent it comes into clinical practice [60]. We believe that by directing this paper outside the society of simulation specialists and instead towards healthcare providers may lead to increased utility and understanding of the advantages of modelling. Moreover, we found the software application to be illustrative and user-friendly for this purpose. The variables included in our model need to be refigured to apply in different hospital settings, which can be achieved by free on-line publication. Hypotheses for interventions could initially be simulated, discarding those that were deemed to have none or little effect. By this mean, proposals of solutions could be selected for clinical trials, thus speeding up the entire research process. Although site-specific data may vary according to guidelines practices and settings, the model in the present form might still be useful for increased understanding and generate hypotheses for hospital infection control practice. Further work is needed to develop and validate the model by retrospectively comparing model estimates with true HCAI data to step-by step to optimize its use.

## Conclusion

This study presents a system dynamic model that is easy to use and aim to capture the complex dynamics of in-hospital transmission of influenza and identify potentially effective interventions to prevent healthcare-associated infections. Our simulations identified antiviral prophylaxis as the most effective way to control in-hospital influenza transmission.

## Supplementary Information

**Additional file 1.** Model equations.

**Additional file 2.** Detailed stock-and-flow diagram with user interface.

**Additional file 3.** xmile version of the model.

## Data Availability

The datasets used during the current study are available from the corresponding author on reasonable request.

## Author’s information

First author MS works as a senior consultant in infectious diseases and infection prevention and control. Her clinical expertise and previous research have focused on outbreak management and nosocomial transmission of respiratory viruses. PH and SH have experience in dynamic modelling. RN is an Ass. Prof in molecular biology. L-MA is an Ass. Professor in Infectious Diseases and JW a Professor in Infectious Diseases.

## References

[1] Leung NHL (2021). Transmissibility and transmission of respiratory viruses. Nat Rev Microbiol.

[2] Stein RA (2011). Super-spreaders in infectious diseases. Int J Infect Dis.

[3] Cassini A, Colzani E, Pini A, Mangen MJ, Plass D, McDonald SA, et al. Impact of infectious diseases on population health using incidence-based disability-adjusted life years (DALYs): results from the Burden of Communicable Diseases in Europe study, European Union and European Economic Area countries, 2009 to 2013. Euro Surveill. 2018;23(16). 10.2807/1560-7917.ES.2018.23.16.17-00454 PMC5915974 29692315

[4] Salgado CD, Farr BM, Hall KK, Hayden FG (2002). Influenza in the acute hospital setting. Lancet Infect Dis.

[5] Jhung MA, D'Mello T, Perez A, Aragon D, Bennett NM, Cooper T (2014). Hospital-onset influenza hospitalizations--United States, 2010–2011. Am J Infect Control.

[6] Cowling BJ, Ip DK, Fang VJ, Suntarattiwong P, Olsen SJ, Levy J (2013). Aerosol transmission is an important mode of influenza A virus spread. Nat Commun.

[7] Tellier R (2009). Aerosol transmission of influenza A virus: a review of new studies. J R Soc Interface.

[8] Vanhems P, Benet T, Munier-Marion E (2016). Nosocomial influenza: encouraging insights and future challenges. Curr Opin Infect Dis.

[9] Voirin N, Barret B, Metzger MH, Vanhems P (2009). Hospital-acquired influenza: a synthesis using the Outbreak Reports and Intervention Studies of Nosocomial Infection (ORION) statement. J Hosp Infect.

[10] Lee BY, Wong KF, Bartsch SM, Yilmaz SL, Avery TR, Brown ST (2013). The Regional Healthcare Ecosystem Analyst (RHEA): a simulation modeling tool to assist infectious disease control in a health system. J Am Med Inform Assoc.

[11] Sterman J Business Dynamics: McGraw-Hill; 2000.

[12] Davahli MR, Karwowski W, Taiar R. A System Dynamics Simulation Applied to Healthcare: A Systematic Review. Int J Environ Res Public Health. 2020;17(16). 10.3390/ijerph17165741 PMC7460395 32784439

[13] Homer JB, Hirsch GB (2006). System dynamics modeling for public health: background and opportunities. Am J Public Health.

[14] Hirsch G, Homer J, Evans E, Zielinski A (2010). A system dynamics model for planning cardiovascular disease interventions. Am J Public Health.

[15] Claeson M, Hallberg S, Holmstrom P, Wennberg AM, Gonzalez H, Paoli J (2016). Modelling the Future: System Dynamics in the Cutaneous Malignant Melanoma Care Pathway. Acta Derm Venereol.

[16] Yu Z, Deng M, Peng C, Song X, Chen Y, Zhang X (2019). A system dynamics modelling simulation based on a cohort of hepatitis B epidemic research in east China community. Epidemiol Infect.

[17] Nguyen LKN, Megiddo I, Howick S (2020). Simulation models for transmission of health care-associated infection: A systematic review. Am J Infect Control.

[18] Cooper K. 2019 [Available from: https://systemdynamics.org/six-reasons-to-apply-system-dynamics-modeling-in-medical-research/.

[19] Ferrer J, Boelle PY, Salomon J, Miliani K, L'Heriteau F, Astagneau P (2014). Management of nurse shortage and its impact on pathogen dissemination in the intensive care unit. Epidemics..

[20] Sadsad R, Sintchenko V, McDonnell GD, Gilbert GL (2013). Effectiveness of hospital-wide methicillin-resistant Staphylococcus aureus (MRSA) infection control policies differs by ward specialty. PLoS One.

[21] Kardas-Sloma L, Boelle PY, Opatowski L, Guillemot D, Temime L (2013). Antibiotic reduction campaigns do not necessarily decrease bacterial resistance: the example of methicillin-resistant Staphylococcus aureus. Antimicrob Agents Chemother.

[22] Wang L, Ruan S (2017). Modeling Nosocomial Infections of Methicillin-Resistant Staphylococcus aureus with Environment Contamination<sup/>. Sci Rep.

[23] van den Dool C, Bonten MJ, Hak E, Wallinga J (2009). Modeling the effects of influenza vaccination of health care workers in hospital departments. Vaccine..

[24] van den Dool C, Bonten MJ, Hak E, Heijne JC, Wallinga J (2008). The effects of influenza vaccination of health care workers in nursing homes: insights from a mathematical model. PLoS Med.

[25] Blanco N, Eisenberg MC, Stillwell T, Foxman B (2016). What Transmission Precautions Best Control Influenza Spread in a Hospital?. Am J Epidemiol.

[26] Wendelboe AM, Grafe C, McCumber M, Anderson MP (2015). Inducing Herd Immunity against Seasonal Influenza in Long-Term Care Facilities through Employee Vaccination Coverage: A Transmission Dynamics Model. Comput Math Methods Med.

[27] Morgan JS, Howick S, Belton V (2017). A toolkit of designs for mixing Discrete Event Simulation and System Dynamics. Eur J Oper Res.

[28] Gunal MM (2012). A guide for building hospital simulation models. Health Syst.

[29] Roberts SD, editor Tutorial on the simulation of healthcare systems2011: IEEE.

[30] Osgood N. Using Traditional and Agent-Based Toolsets for System Dynamics. Proceedings of the (2007). International Conference of the System Dynamics Society; Boston.

[31] Sansone M, Andersson M, Gustavsson L, Andersson LM, Norden R, Westin J. Extensive hospital in-ward clustering revealed by molecular characterization of influenza A virus infection. Clin Infect Dis. 2020. 10.1093/cid/ciaa108 32011654

[32] Chaves SS, Lynfield R, Lindegren ML, Bresee J, Finelli L (2015). The US Influenza Hospitalization Surveillance Network. Emerg Infect Dis.

[33] Lee N, Chan PK, Choi KW, Lui G, Wong B, Cockram CS (2007). Factors associated with early hospital discharge of adult influenza patients. Antivir Ther.

[34] Lee N, Chan PK, Hui DS, Rainer TH, Wong E, Choi KW (2009). Viral loads and duration of viral shedding in adult patients hospitalized with influenza. J Infect Dis.

[35] Haque M, Sartelli M, McKimm J, Abu BM (2018). Health care-associated infections - an overview. Infect Drug Resist.

[36] Lau LL, Ip DK, Nishiura H, Fang VJ, Chan KH, Peiris JS (2013). Heterogeneity in viral shedding among individuals with medically attended influenza A virus infection. J Infect Dis.

[37] Tsang TK, Cowling BJ, Fang VJ, Chan KH, Ip DK, Leung GM (2015). Influenza A Virus Shedding and Infectivity in Households. J Infect Dis.

[38] Dobson J, Whitley RJ, Pocock S, Monto AS (2015). Oseltamivir treatment for influenza in adults: a meta-analysis of randomised controlled trials. Lancet..

[39] Jefferson T, Jones MA, Doshi P, Del Mar CB, Hama R, Thompson MJ, et al. Neuraminidase inhibitors for preventing and treating influenza in healthy adults and children. Cochrane Database Syst Rev. 2014;(4):CD008965. 10.1002/14651858.CD008965.pub3 22258996

[40] FoHM. Public Health Agency of Sweden -Current Influenza report 2015/16 [Available from: https://www.folkhalsomyndigheten.se/publicerat-material/publikationsarkiv/i/influenza-in-sweden-2016-2017-season/.

[41] Hergens MP, Baum U, Brytting M, Ikonen N, Haveri A, Wiman A, et al. Mid-season real-time estimates of seasonal influenza vaccine effectiveness in persons 65 years and older in register-based surveillance, Stockholm County, Sweden, and Finland, January 2017. Euro Surveill. 2017;22(8). 10.2807/1560-7917.ES.2017.22.8.30469 PMC5356437 28251891

[42] Cowling BJ, Ip DKM, Fang VJ, Suntarattiwong P, Olsen SJ, Levy J, et al. Aerosol transmission is an important mode of influenza A virus spread. Nature. Communications. 2013;4. 10.1038/ncomms2922 PMC3682679 23736803

[43] Huttunen R, Syrjanen J (2014). Healthcare workers as vectors of infectious diseases. Eur J Clin Microbiol Infect Dis.

[44] Barlas Y (1996). Formal aspects of model validity and validation in system dynamics System Dynamics Review. J Syst Dynamics Soc.

[45] Jefferson T, Jones MA, Doshi P, Del Mar CB, Hama R, Thompson MJ, et al. Neuraminidase inhibitors for preventing and treating influenza in adults and children. Cochrane Database Syst Rev. 2014;(4):CD008965. 10.1002/14651858.CD008965.pub4 PMC6464969 24718923

[46] Sebille V, Chevret S, Valleron AJ (1997). Modeling the spread of resistant nosocomial pathogens in an intensive-care unit. Infect Control Hosp Epidemiol.

[47] Viana J, Brailsford SC, Harindra V, Harper PR (2014). Combining discrete-event simulation and system dynamics in a healthcare setting: A composite model for Chlamydia infection. Eur J Oper Res.

[48] Wong VW, Cowling BJ, Aiello AE (2014). Hand hygiene and risk of influenza virus infections in the community: a systematic review and meta-analysis. Epidemiol Infect.

[49] Killingley B, Nguyen-Van-Tam J (2013). Routes of influenza transmission. Influenza Other Respir Viruses.

[50] Thomas RE, Jefferson T, Lasserson TJ. Influenza vaccination for healthcare workers who care for people aged 60 or older living in long-term care institutions. Cochrane Database Syst Rev. 2016;(6):CD005187. 10.1002/14651858.CD005187.pub5 PMC8504984 27251461

[51] Okoli GN, Otete HE, Beck CR, Nguyen-Van-Tam JS (2014). Use of neuraminidase inhibitors for rapid containment of influenza: a systematic review and meta-analysis of individual and household transmission studies. PLoS One.

[52] Hayden FG, Atmar RL, Schilling M, Johnson C, Poretz D, Paar D (1999). Use of the selective oral neuraminidase inhibitor oseltamivir to prevent influenza. N Engl J Med.

[53] Ye M, Jacobs A, Khan MN, Jaipaul J, Oda J, Johnson M (2016). Evaluation of the use of oseltamivir prophylaxis in the control of influenza outbreaks in long-term care facilities in Alberta, Canada: a retrospective provincial database analysis. BMJ Open.

[54] Peters PH, Gravenstein S, Norwood P, De Bock V, Van Couter A, Gibbens M (2001). Long-term use of oseltamivir for the prophylaxis of influenza in a vaccinated frail older population. J Am Geriatr Soc.

[55] Munier-Marion E, Benet T, Regis C, Lina B, Morfin F, Vanhems P (2016). Hospitalization in double-occupancy rooms and the risk of hospital-acquired influenza: a prospective cohort study. Clin Microbiol Infect.

[56] Uyeki TM, Bernstein HH, Bradley JS, Englund JA, File TM, Fry AM (2019). Clinical Practice Guidelines by the Infectious Diseases Society of America: 2018 Update on Diagnosis, Treatment, Chemoprophylaxis, and Institutional Outbreak Management of Seasonal Influenzaa. Clin Infect Dis.

[57] Ip DKM, Lau LLH, Chan KH, Fang VJ, Leung GM, Peiris MJS (2016). The Dynamic Relationship Between Clinical Symptomatology and Viral Shedding in Naturally Acquired Seasonal and Pandemic Influenza Virus Infections. Clin Infect Dis.

[58] Fick J, Lindberg RH, Tysklind M, Haemig PD, Waldenstrom J, Wallensten A (2007). Antiviral oseltamivir is not removed or degraded in normal sewage water treatment: implications for development of resistance by influenza A virus. PLoS One.

[59] Jarhult JD, Muradrasoli S, Wahlgren J, Soderstrom H, Orozovic G, Gunnarsson G (2011). Environmental levels of the antiviral oseltamivir induce development of resistance mutation H274Y in influenza A/H1N1 virus in mallards. PLoS One.

[60] Currie DJ, Smith C, Jagals P (2018). The application of system dynamics modelling to environmental health decision-making and policy - a scoping review. BMC Public Health.

