# Supplementary material for: System dynamic modelling of healthcare associated influenza -a tool for infection control
Source: BMC Health Serv Res. 2022 May 27;22:709. doi: 10.1186/s12913-022-07959-7 (PMC9136787; doi:10.1186/s12913-022-07959-7)
Supplement: Supplementary file 1 — Additional file 1. Model equations. [file 12913_2022_7959_MOESM1_ESM.docx]

## Model equations

|  | Equation | Properties |
| --- | --- | --- |
| Acc_IS_Relative_Infectivity(t) | Acc_IS_Relative_Infectivity(t - dt) + (Flow_7) * dt | INIT Acc_IS_Relative_Infectivity = 0 |
| Accumulated_HCAI_infected_by_HCAI(t) | Accumulated_HCAI_infected_by_HCAI(t - dt) + (Flow_3) * dt | INIT Accumulated_HCAI_infected_by_HCAI = 0 |
| Accumulated_HCAI_infected_by_Influenza_not_suspected(t) | Accumulated_HCAI_infected_by_Influenza_not_suspected(t - dt) + (Flow_2) * dt | INIT Accumulated_HCAI_infected_by_Influenza_not_suspected = 0 |
| Accumulated_HCAI_infected_by_Influenza_suspected(t) | Accumulated_HCAI_infected_by_Influenza_suspected(t - dt) + (Flow_1) * dt | INIT Accumulated_HCAI_infected_by_Influenza_suspected = 0 |
| "Accumulated_Hospital-associated_influenza_(HCAI)"(t) | "Accumulated_Hospital-associated_influenza_(HCAI)"(t - dt) + (Infected_HCAI) * dt | INIT "Accumulated_Hospital-associated_influenza_(HCAI)" = 0 |
| Accumulated_INS(t) | Accumulated_INS(t - dt) + (Flow_6) * dt | INIT Accumulated_INS = 0 |
| Accumulated_IS(t) | Accumulated_IS(t - dt) + (Flow_5) * dt | INIT Accumulated_IS = 0 |
| HCAI_Day_1(t) | HCAI_Day_1(t - dt) + (HCAI - HCAI_Day_1t2) * dt | INIT HCAI_Day_1 = 0 TRANSIT TIME = 1 DISCRETE ACCEPT MULTIPLE BATCHES |
| HCAI_Day_4(t) | HCAI_Day_4(t - dt) + (HCAI_Day_3t4 - HCAI_Day_4t5) * dt | INIT HCAI_Day_4 = 0 TRANSIT TIME = 1 DISCRETE ACCEPT MULTIPLE BATCHES |
| HCAI_Day_5(t) | HCAI_Day_5(t - dt) + (HCAI_Day_4t5 - HCAI_Day_5t6) * dt | INIT HCAI_Day_5 = 0 TRANSIT TIME = 1 DISCRETE ACCEPT MULTIPLE BATCHES |
| HCAI_Day_6(t) | HCAI_Day_6(t - dt) + (HCAI_Day_5t6 - HCAI_Day_6t7) * dt | INIT HCAI_Day_6 = 0 TRANSIT TIME = 1 DISCRETE ACCEPT MULTIPLE BATCHES |
| HCAI_Day_7(t) | HCAI_Day_7(t - dt) + (HCAI_Day_6t7 - HCAI_Day_7t8) * dt | INIT HCAI_Day_7 = 0 TRANSIT TIME = 1 DISCRETE ACCEPT MULTIPLE BATCHES |
| HCAI_Day_8(t) | HCAI_Day_8(t - dt) + (HCAI_Day_7t8 - HCAI_Day_8_out) * dt | INIT HCAI_Day_8 = 0 TRANSIT TIME = 1 DISCRETE ACCEPT MULTIPLE BATCHES |
| HCAI_Day2(t) | HCAI_Day2(t - dt) + (HCAI_Day_1t2 - HCAI_Day_2t3) * dt | INIT HCAI_Day2 = 0 TRANSIT TIME = 1 DISCRETE ACCEPT MULTIPLE BATCHES |
| INS_Day_2(t) | INS_Day_2(t - dt) + (INS - INS_Day_2t3) * dt | INIT INS_Day_2 = 0 TRANSIT TIME = 1 DISCRETE ACCEPT MULTIPLE BATCHES |
| INS_Day_3(t) | INS_Day_3(t - dt) + (INS_Day_2t3 - INS_Day_3t4) * dt | INIT INS_Day_3 = 0 TRANSIT TIME = 1 DISCRETE ACCEPT MULTIPLE BATCHES |
| INS_Day_4(t) | INS_Day_4(t - dt) + (INS_Day_3t4 - INS_Day_4t5) * dt | INIT INS_Day_4 = 0 TRANSIT TIME = 1 DISCRETE ACCEPT MULTIPLE BATCHES |
| INS_Day_5(t) | INS_Day_5(t - dt) + (INS_Day_4t5 - INS_Day_5t6) * dt | INIT INS_Day_5 = 0 TRANSIT TIME = 1 DISCRETE ACCEPT MULTIPLE BATCHES |
| INS_Day_6(t) | INS_Day_6(t - dt) + (INS_Day_5t6 - INS_Day_6t7) * dt | INIT INS_Day_6 = 0 TRANSIT TIME = 1 DISCRETE ACCEPT MULTIPLE BATCHES |
| INS_Day_7(t) | INS_Day_7(t - dt) + (INS_Day_6t7 - INS_Day_7t8) * dt | INIT INS_Day_7 = 0 TRANSIT TIME = 1 DISCRETE ACCEPT MULTIPLE BATCHES |
| INS_Day_8(t) | INS_Day_8(t - dt) + (INS_Day_7t8 - INS_Day_8_out) * dt | INIT INS_Day_8 = 0 TRANSIT TIME = 1 DISCRETE ACCEPT MULTIPLE BATCHES |
| IS_Day_2(t) | IS_Day_2(t - dt) + (IS - IS_Day_2t3) * dt | INIT IS_Day_2 = 0 TRANSIT TIME = 1 DISCRETE ACCEPT MULTIPLE BATCHES |
| IS_Day_3(t) | IS_Day_3(t - dt) + (IS_Day_2t3 - IS_Day_3t4) * dt | INIT IS_Day_3 = 0 TRANSIT TIME = 1 DISCRETE ACCEPT MULTIPLE BATCHES |
| IS_Day_4(t) | IS_Day_4(t - dt) + (IS_Day_3t4 - IS_Day_4t5) * dt | INIT IS_Day_4 = 0 TRANSIT TIME = 1 DISCRETE ACCEPT MULTIPLE BATCHES |
| IS_Day_5(t) | IS_Day_5(t - dt) + (IS_Day_4t5 - IS_Day_5t6) * dt | INIT IS_Day_5 = 0 TRANSIT TIME = 1 DISCRETE ACCEPT MULTIPLE BATCHES |
| IS_Day_6(t) | IS_Day_6(t - dt) + (IS_Day_5t6 - IS_Day_6t7) * dt | INIT IS_Day_6 = 0 TRANSIT TIME = 1 DISCRETE ACCEPT MULTIPLE BATCHES |
| IS_Day_7(t) | IS_Day_7(t - dt) + (IS_Day_6t7 - IS_Day_7t8) * dt | INIT IS_Day_7 = 0 TRANSIT TIME = 1 DISCRETE ACCEPT MULTIPLE BATCHES |
| IS_Day_8(t) | IS_Day_8(t - dt) + (IS_Day_7t8 - IS_Day_8_out) * dt | INIT IS_Day_8 = 0 TRANSIT TIME = 1 DISCRETE ACCEPT MULTIPLE BATCHES |
| Not_influenza(t) | Not_influenza(t - dt) + (From_Emergency_not_influenza - Not_influensa_leaving - HCAI) * dt | INIT Not_influenza = 135 |
| SU_Dag_3(t) | SU_Dag_3(t - dt) + (HCAI_Day_2t3 - HCAI_Day_3t4) * dt | INIT SU_Dag_3 = 0 TRANSIT TIME = 1 DISCRETE ACCEPT MULTIPLE BATCHES |
| Total_HCAI(t) | Total_HCAI(t - dt) + (HCAI_Day_8_out) * dt | INIT Total_HCAI = 0 |
| Total_INS(t) | Total_INS(t - dt) + (INS_Day_8_out) * dt | INIT Total_INS = 0 |
| Total_IS(t) | Total_IS(t - dt) + (IS_Day_8_out) * dt | INIT Total_IS = 0 |
| Flow_1 | Not_influenza * Calibration_factor/10* ((1-"Ratio_non-flu_receiving_prophylactic_medication_(%)"/100) + ("Ratio_non-flu_receiving_prophylactic_medication_(%)"/100) *(1-"Prophylactic_effectiveness_(%)"/100)) * Average_#_other_exposed_patients_in_shared_rooms * ( (1-"Vaccination_coverage_(%)"/100)+ (("Vaccination_coverage_(%)"/100)*(1-"Vaccine_effectiveness_(%)"/100)) ) * ( ( IS_Relative_Infectivity) / (HCAI_Relative_Infectivity+IS_Relative_Infectivity+INS_Relative_infectivity+ Not_influenza) ) |  |
| Flow_2 | Not_influenza * Calibration_factor/10* ((1-"Ratio_non-flu_receiving_prophylactic_medication_(%)"/100) + ("Ratio_non-flu_receiving_prophylactic_medication_(%)"/100) *(1-"Prophylactic_effectiveness_(%)"/100)) * Average_#_other_exposed_patients_in_shared_rooms * ( (1-"Vaccination_coverage_(%)"/100)+ (("Vaccination_coverage_(%)"/100)*(1-"Vaccine_effectiveness_(%)"/100)) ) * ( ( INS_Relative_infectivity) / (HCAI_Relative_Infectivity+IS_Relative_Infectivity+INS_Relative_infectivity+ Not_influenza) ) |  |
| Flow_3 | Not_influenza * Calibration_factor/10* ((1-"Ratio_non-flu_receiving_prophylactic_medication_(%)"/100) + ("Ratio_non-flu_receiving_prophylactic_medication_(%)"/100) *(1-"Prophylactic_effectiveness_(%)"/100)) * Average_#_other_exposed_patients_in_shared_rooms * ( (1-"Vaccination_coverage_(%)"/100)+ (("Vaccination_coverage_(%)"/100)*(1-"Vaccine_effectiveness_(%)"/100)) ) * ( (HCAI_Relative_Infectivity) / (HCAI_Relative_Infectivity+IS_Relative_Infectivity+INS_Relative_infectivity+ Not_influenza) ) |  |
| Flow_5 | IS |  |
| Flow_6 | INS |  |
| Flow_7 | IS_Relative_Infectivity |  |
| From_Emergency_not_influenza | Total_ED_arrivals_per_year/365 -IS-INS |  |
| HCAI | Not_influenza * Calibration_factor/10* ((1-"Ratio_non-flu_receiving_prophylactic_medication_(%)"/100) + ("Ratio_non-flu_receiving_prophylactic_medication_(%)"/100) *(1-"Prophylactic_effectiveness_(%)"/100)) * Average_#_other_exposed_patients_in_shared_rooms * ( (1-"Vaccination_coverage_(%)"/100)+ (("Vaccination_coverage_(%)"/100)*(1-"Vaccine_effectiveness_(%)"/100)) ) * ( (HCAI_Relative_Infectivity+ IS_Relative_Infectivity+ INS_Relative_infectivity) / (HCAI_Relative_Infectivity+IS_Relative_Infectivity+INS_Relative_infectivity+ Not_influenza) ) | OUTFLOW PRIORITY: 2 |
| HCAI_Day_1t2 | CONVEYOR OUTFLOW |  |
| HCAI_Day_2t3 | CONVEYOR OUTFLOW |  |
| HCAI_Day_3t4 | CONVEYOR OUTFLOW |  |
| HCAI_Day_4t5 | CONVEYOR OUTFLOW |  |
| HCAI_Day_5t6 | CONVEYOR OUTFLOW |  |
| HCAI_Day_6t7 | CONVEYOR OUTFLOW |  |
| HCAI_Day_7t8 | CONVEYOR OUTFLOW |  |
| HCAI_Day_8_out | CONVEYOR OUTFLOW |  |
| Infected_HCAI | HCAI |  |
| INS | (1-"Diagnostic_accuracy_at_Emergency_(%)"/100)* Influenza_season* Total_#_influenza_patients_from_Emergency |  |
| INS_Day_2t3 | CONVEYOR OUTFLOW |  |
| INS_Day_3t4 | CONVEYOR OUTFLOW |  |
| INS_Day_4t5 | CONVEYOR OUTFLOW |  |
| INS_Day_5t6 | CONVEYOR OUTFLOW |  |
| INS_Day_6t7 | CONVEYOR OUTFLOW |  |
| INS_Day_7t8 | CONVEYOR OUTFLOW |  |
| INS_Day_8_out | CONVEYOR OUTFLOW |  |
| IS | ("Diagnostic_accuracy_at_Emergency_(%)"/100)*Influenza_season* Total_#_influenza_patients_from_Emergency |  |
| IS_Day_2t3 | CONVEYOR OUTFLOW |  |
| IS_Day_3t4 | CONVEYOR OUTFLOW |  |
| IS_Day_4t5 | CONVEYOR OUTFLOW |  |
| IS_Day_5t6 | CONVEYOR OUTFLOW |  |
| IS_Day_6t7 | CONVEYOR OUTFLOW |  |
| IS_Day_7t8 | CONVEYOR OUTFLOW |  |
| IS_Day_8_out | CONVEYOR OUTFLOW |  |
| Not_influensa_leaving | Not_influenza/"Care_time,_not_influenza" | OUTFLOW PRIORITY: 1 |
| Average_#_other_exposed_patients_in_shared_rooms | 2 |  |
| "Average_viral_titre_level_treated_day_1-8_(should_be_1,00)" | MEAN("Viral_titre_level_day_1_(untreated)"; "Viral_titre_level_day_2_(untreated)"; "Viral_titre_level_day_3_(untreated)"; "Viral_titre_level_day_4_(untreated)"; "Viral_titre_level_day_5_(untreated)"; "Viral_titre_level_day_6_(untreated)"; "Viral_titre_level_day_7_(untreated)"; "Viral_titre_level_day_8_(untreated)") |  |
| Calibration_factor | ,001 |  |
| "Care_time,_not_influenza" | 5 |  |
| "Diagnostic_accuracy_at_Emergency_(%)" | 60 |  |
| HCAI_Infectvity_Day_1 | ((("Share_of_HCAI_treated_day_2_(%)"/100)*"Viral_titre_level_day_1_(treated)")+ ((1-("Share_of_HCAI_treated_day_2_(%)" /100))*"Viral_titre_level_day_1_(untreated)")) |  |
| HCAI_Infectvity_Day_2 | ((("Share_of_HCAI_treated_day_2_(%)"/100)*"Viral_titre_level_day_2_(treated)")+ ((1-("Share_of_HCAI_treated_day_2_(%)" /100))*"Viral_titre_level_day_2_(untreated)")) |  |
| HCAI_Infectvity_Day_3 | ((("Share_of_HCAI_treated_day_2_(%)"/100)*"Viral_titre_level_day_3_(treated)")+ ((1-("Share_of_HCAI_treated_day_2_(%)" /100))*"Viral_titre_level_day_3_(untreated)")) |  |
| HCAI_Infectvity_Day_4 | ((("Share_of_HCAI_treated_day_2_(%)"/100)*"Viral_titre_level_day_4_(treated)")+ ((1-("Share_of_HCAI_treated_day_2_(%)" /100))*"Viral_titre_level_day_4_(untreated)")) |  |
| HCAI_Infectvity_Day_5 | ((("Share_of_HCAI_treated_day_2_(%)"/100)*"Viral_titre_level_day_5_(treated)")+ ((1-("Share_of_HCAI_treated_day_2_(%)" /100))*"Viral_titre_level_day_5_(untreated)")) |  |
| HCAI_Infectvity_Day_6 | ((("Share_of_HCAI_treated_day_2_(%)"/100)*"Viral_titre_level_day_6_(treated)")+ ((1-("Share_of_HCAI_treated_day_2_(%)" /100))*"Viral_titre_level_day_6_(untreated)")) |  |
| HCAI_Infectvity_Day_7 | ((("Share_of_HCAI_treated_day_2_(%)"/100)*"Viral_titre_level_day_7_(treated)")+ ((1-("Share_of_HCAI_treated_day_2_(%)" /100))*"Viral_titre_level_day_7_(untreated)")) |  |
| HCAI_Infectvity_Day_8 | ((("Share_of_HCAI_treated_day_2_(%)"/100)*"Viral_titre_level_day_8_(treated)")+ ((1-("Share_of_HCAI_treated_day_2_(%)" /100))*"Viral_titre_level_day_8_(untreated)")) |  |
| HCAI_Relative_Infectivity | ((HCAI_Day_1*HCAI_Infectvity_Day_1)+(HCAI_Day2*HCAI_Infectvity_Day_2)+(SU_Dag_3*HCAI_Infectvity_Day_3)+(HCAI_Day_4*HCAI_Infectvity_Day_4)+(HCAI_Day_5*HCAI_Infectvity_Day_5)+(HCAI_Day_6*HCAI_Infectvity_Day_6)+(HCAI_Day_7*HCAI_Infectvity_Day_7)+(HCAI_Day_8*HCAI_Infectvity_Day_8)) |  |
| HCAI_sum | HCAI_Day_1+HCAI_Day2+SU_Dag_3+HCAI_Day_4+HCAI_Day_5+HCAI_Day_6+HCAI_Day_7+HCAI_Day_8 |  |
| Influenza_season | (S1819*"Season_18--19"+S1718*"Season_17--18"+S1617*"Season_16--17"+S1516*"Season_15--16"+S1415*"Season_14--15"+S1314*"Season_13--14")/100 |  |
| INS_infectivity_day_2 | ((("Share_of_INS_treated_day_2_(%)"/100)*"Viral_titre_level_day_2_(treated)")+ ((1-("Share_of_INS_treated_day_2_(%)"/100))*"Viral_titre_level_day_2_(untreated)")) |  |
| INS_infectivity_day_3 | ((("Share_of_INS_treated_day_2_(%)"/100)*"Viral_titre_level_day_3_(treated)")+ ((1-("Share_of_INS_treated_day_2_(%)"/100))*"Viral_titre_level_day_3_(untreated)")) |  |
| INS_infectivity_day_4 | ((("Share_of_INS_treated_day_2_(%)"/100)*"Viral_titre_level_day_4_(treated)")+ ((1-("Share_of_INS_treated_day_2_(%)"/100))*"Viral_titre_level_day_4_(untreated)")) |  |
| INS_infectivity_day_5 | ((("Share_of_INS_treated_day_2_(%)"/100)*"Viral_titre_level_day_5_(treated)")+ ((1-("Share_of_INS_treated_day_2_(%)"/100))*"Viral_titre_level_day_5_(untreated)")) |  |
| INS_infectivity_day_6 | ((("Share_of_INS_treated_day_2_(%)"/100)*"Viral_titre_level_day_6_(treated)")+ ((1-("Share_of_INS_treated_day_2_(%)"/100))*"Viral_titre_level_day_6_(untreated)")) |  |
| INS_infectivity_day_7 | ((("Share_of_INS_treated_day_2_(%)"/100)*"Viral_titre_level_day_7_(treated)")+ ((1-("Share_of_INS_treated_day_2_(%)"/100))*"Viral_titre_level_day_7_(untreated)")) |  |
| INS_infectivity_day_8 | ((("Share_of_INS_treated_day_2_(%)"/100)*"Viral_titre_level_day_8_(treated)")+ ((1-("Share_of_INS_treated_day_2_(%)"/100))*"Viral_titre_level_day_8_(untreated)")) |  |
| INS_Relative_infectivity | ((INS_Day_2*INS_infectivity_day_2)+(INS_Day_3*INS_infectivity_day_3)+(INS_Day_4*INS_infectivity_day_4)+(INS_Day_5*INS_infectivity_day_5)+(INS_Day_6*INS_infectivity_day_6)+(INS_Day_7*INS_infectivity_day_7)+(INS_Day_8*INS_infectivity_day_8)) |  |
| INS_sum | INS_Day_2+INS_Day_3+INS_Day_4+INS_Day_5+INS_Day_6+INS_Day_7+INS_Day_8 |  |
| IS_Infectivity_Day_2 | ((("Share_of_IS_treated_day_2_(%)"/100)*"Viral_titre_level_day_2_(treated)")+ ((1-("Share_of_IS_treated_day_2_(%)"/100))*"Viral_titre_level_day_2_(untreated)")) |  |
| IS_Infectivity_Day_3 | ((("Share_of_IS_treated_day_2_(%)"/100)*"Viral_titre_level_day_3_(treated)")+ ((1-("Share_of_IS_treated_day_2_(%)"/100))*"Viral_titre_level_day_3_(untreated)")) |  |
| IS_Infectivity_Day_4 | ((("Share_of_IS_treated_day_2_(%)"/100)*"Viral_titre_level_day_4_(treated)")+ ((1-("Share_of_IS_treated_day_2_(%)"/100))*"Viral_titre_level_day_4_(untreated)")) |  |
| IS_Infectivity_Day_5 | ((("Share_of_IS_treated_day_2_(%)"/100)*"Viral_titre_level_day_5_(treated)")+ ((1-("Share_of_IS_treated_day_2_(%)"/100))*"Viral_titre_level_day_5_(untreated)")) |  |
| IS_Infectivity_Day_6 | ((("Share_of_IS_treated_day_2_(%)"/100)*"Viral_titre_level_day_6_(treated)")+ ((1-("Share_of_IS_treated_day_2_(%)"/100))*"Viral_titre_level_day_6_(untreated)")) |  |
| IS_Infectivity_Day_7 | ((("Share_of_IS_treated_day_2_(%)"/100)*"Viral_titre_level_day_7_(treated)")+ ((1-("Share_of_IS_treated_day_2_(%)"/100))*"Viral_titre_level_day_7_(untreated)")) |  |
| IS_Infectivity_Day_8 | ((("Share_of_IS_treated_day_2_(%)"/100)*"Viral_titre_level_day_8_(treated)")+ ((1-("Share_of_IS_treated_day_2_(%)"/100))*"Viral_titre_level_day_8_(untreated)")) |  |
| IS_Relative_Infectivity | ((IS_Day_2*IS_Infectivity_Day_2)+ (IS_Day_3*IS_Infectivity_Day_3)+(IS_Day_4*IS_Infectivity_Day_4)+(IS_Day_5*IS_Infectivity_Day_5)+(IS_Day_6*IS_Infectivity_Day_6)+(IS_Day_7*IS_Infectivity_Day_7)+(IS_Day_8*IS_Infectivity_Day_8)) |  |
| IS_sum | IS_Day_2+IS_Day_3+IS_Day_4+IS_Day_5+IS_Day_6+IS_Day_7+IS_Day_8 |  |
| "Prophylactic_effectiveness_(%)" | 80 |  |
| "Ratio_infected_-_non-infected" | 100*HCAI/From_Emergency_not_influenza |  |
| "Ratio_non-flu_receiving_prophylactic_medication_(%)" | 30 |  |
| S1314 | 0 |  |
| S1415 | 0 |  |
| S1516 | 0 |  |
| S1617 | 0 |  |
| S1718 | 1 |  |
| S1819 | 1 |  |
| "Season_13--14" | GRAPH(TIME) Points: (1,0, 0,011261895377), (2,0, 0,0104574742786), (3,0, 0,00965305318028), (4,0, 0,00884863208192), (5,0, 0,00804421098357), (6,0, 0,00723978988521), (7,0, 0,00643536878685), (8,0, 0,0056309476885), (9,0, 0,0056309476885), (10,0, 0,0056309476885), (11,0, 0,0056309476885), (12,0, 0,0056309476885), (13,0, 0,0056309476885), (14,0, 0,0056309476885), (15,0, 0,0056309476885), (16,0, 0,00643536878685), (17,0, 0,00723978988521), (18,0, 0,00804421098357), (19,0, 0,00884863208192), (20,0, 0,00965305318028), (21,0, 0,0104574742786), (22,0, 0,011261895377), (23,0, 0,0144795797704), (24,0, 0,0176972641638), (25,0, 0,0209149485573), (26,0, 0,0241326329507), (27,0, 0,0273503173441), (28,0, 0,0305680017375), (29,0, 0,033785686131), (30,0, 0,0329812650326), (31,0, 0,0321768439343), (32,0, 0,0313724228359), (33,0, 0,0305680017375), (34,0, 0,0297635806392), (35,0, 0,0289591595408), (36,0, 0,0281547384425), (37,0, 0,0313724228359), (38,0, 0,0345901072293), (39,0, 0,0378077916228), (40,0, 0,0410254760162), (41,0, 0,0442431604096), (42,0, 0,047460844803), (43,0, 0,0506785291965), (44,0, 0,0458520026063), (45,0, 0,0410254760162), (46,0, 0,036198949426), (47,0, 0,0313724228359), (48,0, 0,0265458962458), (49,0, 0,0217193696556), (50,0, 0,0168928430655), (51,0, 0,0193061063606), (52,0, 0,0217193696556), (53,0, 0,0241326329507), (54,0, 0,0265458962458), (55,0, 0,0289591595408), (56,0, 0,0313724228359), (57,0, 0,033785686131), (58,0, 0,0402210549178), (59,0, 0,0466564237047), (60,0, 0,0530917924915), (61,0, 0,0595271612784), (62,0, 0,0659625300652), (63,0, 0,0723978988521), (64,0, 0,0788332676389), (65,0, 0,0868774786225), (66,0, 0,0949216896061), (67,0, 0,10296590059), (68,0, 0,111010111573), (69,0, 0,119054322557), (70,0, 0,12709853354), (71,0, 0,135142744524), (72,0, 0,177777062737), (73,0, 0,22041138095), (74,0, 0,263045699163), (75,0, 0,305680017375), (76,0, 0,348314335588), (77,0, 0,390948653801), (78,0, 0,433582972014), (79,0, 0,440018340801), (80,0, 0,446453709588), (81,0, 0,452889078375), (82,0, 0,459324447162), (83,0, 0,465759815948), (84,0, 0,472195184735), (85,0, 0,478630553522), (86,0, 0,483457080112), (87,0, 0,488283606702), (88,0, 0,493110133293), (89,0, 0,497936659883), (90,0, 0,502763186473), (91,0, 0,507589713063), (92,0, 0,512416239653), (93,0, 0,516438345145), (94,0, 0,520460450637), (95,0, 0,524482556128), (96,0, 0,52850466162), (97,0, 0,532526767112), (98,0, 0,536548872604), (99,0, 0,540570978096), (100,0, 0,518047187342), (101,0, 0,495523396588), (102,0, 0,472999605834), (103,0, 0,45047581508), (104,0, 0,427952024326), (105,0, 0,405428233572), (106,0, 0,382904442818), (107,0, 0,402210549178), (108,0, 0,421516655539), (109,0, 0,440822761899), (110,0, 0,46012886826), (111,0, 0,479434974621), (112,0, 0,498741080981), (113,0, 0,518047187342), (114,0, 0,551028452374), (115,0, 0,584009717407), (116,0, 0,616990982439), (117,0, 0,649972247472), (118,0, 0,682953512505), (119,0, 0,715934777537), (120,0, 0,74891604257), (121,0, 0,820509520324), (122,0, 0,892102998077), (123,0, 0,963696475831), (124,0, 1,03528995358), (125,0, 1,10688343134), (126,0, 1,17847690909), (127,0, 1,25007038685), (128,0, 1,28868259957), (129,0, 1,32729481229), (130,0, 1,36590702501), (131,0, 1,40451923773), (132,0, 1,44313145045), (133,0, 1,48174366317), (134,0, 1,52035587589), (135,0, 1,55172829873), (136,0, 1,58310072157), (137,0, 1,6144731444), (138,0, 1,64584556724), (139,0, 1,67721799007), (140,0, 1,70859041291), (141,0, 1,73996283575), (142,0, 1,67802241117), (143,0, 1,6160819866), (144,0, 1,55414156202), (145,0, 1,49220113745), (146,0, 1,43026071288), (147,0, 1,3683202883), (148,0, 1,30637986373), (149,0, 1,2742030198), (150,0, 1,24202617586), (151,0, 1,20984933193), (152,0, 1,17767248799), (153,0, 1,14549564406), (154,0, 1,11331880013), (155,0, 1,08114195619), (156,0, 1,04735627006), (157,0, 1,01357058393), (158,0, 0,979784897798), (159,0, 0,945999211667), (160,0, 0,912213525536), (161,0, 0,878427839405), (162,0, 0,844642153274), (163,0, 0,808443203848), (164,0, 0,772244254422), (165,0, 0,736045304996), (166,0, 0,69984635557), (167,0, 0,663647406144), (168,0, 0,627448456718), (169,0, 0,591249507292), (170,0, 0,575965506423), (171,0, 0,560681505555), (172,0, 0,545397504686), (173,0, 0,530113503817), (174,0, 0,514829502948), (175,0, 0,499545502079), (176,0, 0,484261501211), (177,0, 0,471390763637), (178,0, 0,458520026063), (179,0, 0,44564928849), (180,0, 0,432778550916), (181,0, 0,419907813342), (182,0, 0,407037075768), (183,0, 0,394166338195), (184,0, 0,398992864785), (185,0, 0,403819391375), (186,0, 0,408645917965), (187,0, 0,413472444555), (188,0, 0,418298971145), (189,0, 0,423125497736), (190,0, 0,427952024326), (191,0, 0,403819391375), (192,0, 0,379686758424), (193,0, 0,355554125474), (194,0, 0,331421492523), (195,0, 0,307288859572), (196,0, 0,283156226622), (197,0, 0,259023593671), (198,0, 0,259023593671), (199,0, 0,259023593671), (200,0, 0,259023593671), (201,0, 0,259023593671), (202,0, 0,259023593671), (203,0, 0,259023593671), (204,0, 0,259023593671), (205,0, 0,236499802917), (206,0, 0,213976012163), (207,0, 0,191452221409), (208,0, 0,168928430655), (209,0, 0,146404639901), (210,0, 0,123880849147), (211,0, 0,101357058393), (212,0, 0,0868774786225), (213,0, 0,0723978988521), (214,0, 0,0579183190817), (215,0, 0,0434387393113), (216,0, 0,0289591595408), (217,0, 0,0144795797704), (218,0, 0,000), (219,0, 0,000), (220,0, 0,000), (221,0, 0,000), (222,0, 0,000), (223,0, 0,000), (224,0, 0,000), (225,0, 0,000) |  |
| "Season_14--15" | GRAPH(TIME) Points: (1,0, 0,000), (2,0, 0,000238859588779), (3,0, 0,000477719177559), (4,0, 0,000716578766338), (5,0, 0,000955438355117), (6,0, 0,0011942979439), (7,0, 0,00143315753268), (8,0, 0,00167201712146), (9,0, 0,00167201712146), (10,0, 0,00167201712146), (11,0, 0,00167201712146), (12,0, 0,00167201712146), (13,0, 0,00167201712146), (14,0, 0,00167201712146), (15,0, 0,00167201712146), (16,0, 0,00214973629901), (17,0, 0,00262745547657), (18,0, 0,00310517465413), (19,0, 0,00358289383169), (20,0, 0,00406061300925), (21,0, 0,00453833218681), (22,0, 0,00501605136437), (23,0, 0,00525491095315), (24,0, 0,00549377054192), (25,0, 0,0057326301307), (26,0, 0,00597148971948), (27,0, 0,00621034930826), (28,0, 0,00644920889704), (29,0, 0,00668806848582), (30,0, 0,00716578766338), (31,0, 0,00764350684094), (32,0, 0,0081212260185), (33,0, 0,00859894519606), (34,0, 0,00907666437361), (35,0, 0,00955438355117), (36,0, 0,0100321027287), (37,0, 0,0100321027287), (38,0, 0,0100321027287), (39,0, 0,0100321027287), (40,0, 0,0100321027287), (41,0, 0,0100321027287), (42,0, 0,0100321027287), (43,0, 0,0100321027287), (44,0, 0,00955438355117), (45,0, 0,00907666437361), (46,0, 0,00859894519606), (47,0, 0,0081212260185), (48,0, 0,00764350684094), (49,0, 0,00716578766338), (50,0, 0,00668806848582), (51,0, 0,00788236642972), (52,0, 0,00907666437361), (53,0, 0,0102709623175), (54,0, 0,0114652602614), (55,0, 0,0126595582053), (56,0, 0,0138538561492), (57,0, 0,0150481540931), (58,0, 0,0148092945043), (59,0, 0,0145704349155), (60,0, 0,0143315753268), (61,0, 0,014092715738), (62,0, 0,0138538561492), (63,0, 0,0136149965604), (64,0, 0,0133761369716), (65,0, 0,014092715738), (66,0, 0,0148092945043), (67,0, 0,0155258732707), (68,0, 0,016242452037), (69,0, 0,0169590308033), (70,0, 0,0176756095697), (71,0, 0,018392188336), (72,0, 0,0234082397004), (73,0, 0,0284242910647), (74,0, 0,0334403424291), (75,0, 0,0384563937935), (76,0, 0,0434724451578), (77,0, 0,0484884965222), (78,0, 0,0535045478866), (79,0, 0,0563708629519), (80,0, 0,0592371780173), (81,0, 0,0621034930826), (82,0, 0,064969808148), (83,0, 0,0678361232133), (84,0, 0,0707024382787), (85,0, 0,073568753344), (86,0, 0,0752407704655), (87,0, 0,0769127875869), (88,0, 0,0785848047084), (89,0, 0,0802568218299), (90,0, 0,0819288389513), (91,0, 0,0836008560728), (92,0, 0,0852728731942), (93,0, 0,103187342353), (94,0, 0,121101811511), (95,0, 0,13901628067), (96,0, 0,156930749828), (97,0, 0,174845218986), (98,0, 0,192759688145), (99,0, 0,210674157303), (100,0, 0,225722311396), (101,0, 0,24077046549), (102,0, 0,255818619583), (103,0, 0,270866773676), (104,0, 0,285914927769), (105,0, 0,300963081862), (106,0, 0,316011235955), (107,0, 0,32293816403), (108,0, 0,329865092104), (109,0, 0,336792020179), (110,0, 0,343718948253), (111,0, 0,350645876328), (112,0, 0,357572804403), (113,0, 0,364499732477), (114,0, 0,386952533823), (115,0, 0,409405335168), (116,0, 0,431858136513), (117,0, 0,454310937858), (118,0, 0,476763739204), (119,0, 0,499216540549), (120,0, 0,521669341894), (121,0, 0,574935030192), (122,0, 0,62820071849), (123,0, 0,681466406787), (124,0, 0,734732095085), (125,0, 0,787997783383), (126,0, 0,841263471681), (127,0, 0,894529159979), (128,0, 0,964276159902), (129,0, 1,03402315983), (130,0, 1,10377015975), (131,0, 1,17351715967), (132,0, 1,2432641596), (133,0, 1,31301115952), (134,0, 1,38275815944), (135,0, 1,41166016969), (136,0, 1,44056217993), (137,0, 1,46946419017), (138,0, 1,49836620041), (139,0, 1,52726821066), (140,0, 1,5561702209), (141,0, 1,58507223114), (142,0, 1,57289039211), (143,0, 1,56070855308), (144,0, 1,54852671406), (145,0, 1,53634487503), (146,0, 1,524163036), (147,0, 1,51198119697), (148,0, 1,49979935795), (149,0, 1,49191699152), (150,0, 1,48403462509), (151,0, 1,47615225866), (152,0, 1,46826989223), (153,0, 1,4603875258), (154,0, 1,45250515937), (155,0, 1,44462279294), (156,0, 1,41070473133), (157,0, 1,37678666972), (158,0, 1,34286860812), (159,0, 1,30895054651), (160,0, 1,2750324849), (161,0, 1,2411144233), (162,0, 1,20719636169), (163,0, 1,16037988229), (164,0, 1,11356340289), (165,0, 1,06674692349), (166,0, 1,01993044409), (167,0, 0,973113964687), (168,0, 0,926297485286), (169,0, 0,879481005886), (170,0, 0,883063899717), (171,0, 0,886646793549), (172,0, 0,890229687381), (173,0, 0,893812581212), (174,0, 0,897395475044), (175,0, 0,900978368876), (176,0, 0,904561262707), (177,0, 0,882347320951), (178,0, 0,860133379194), (179,0, 0,837919437438), (180,0, 0,815705495681), (181,0, 0,793491553925), (182,0, 0,771277612168), (183,0, 0,749063670412), (184,0, 0,761962088206), (185,0, 0,774860506), (186,0, 0,787758923794), (187,0, 0,800657341588), (188,0, 0,813555759382), (189,0, 0,826454177176), (190,0, 0,839352594971), (191,0, 0,792774975159), (192,0, 0,746197355347), (193,0, 0,699619735535), (194,0, 0,653042115723), (195,0, 0,606464495911), (196,0, 0,559886876099), (197,0, 0,513309256287), (198,0, 0,503516013147), (199,0, 0,493722770007), (200,0, 0,483929526867), (201,0, 0,474136283727), (202,0, 0,464343040587), (203,0, 0,454549797447), (204,0, 0,444756554307), (205,0, 0,413704807766), (206,0, 0,382653061224), (207,0, 0,351601314683), (208,0, 0,320549568142), (209,0, 0,289497821601), (210,0, 0,258446075059), (211,0, 0,227394328518), (212,0, 0,194909424444), (213,0, 0,16242452037), (214,0, 0,129939616296), (215,0, 0,097454712222), (216,0, 0,064969808148), (217,0, 0,032484904074), (218,0, 0,000), (219,0, 0,000), (220,0, 0,000), (221,0, 0,000), (222,0, 0,000), (223,0, 0,000), (224,0, 0,000), (225,0, 0,000) |  |
| "Season_15--16" | GRAPH(TIME) Points: (1,0, 0,00165152766309), (2,0, 0,00259525775628), (3,0, 0,00353898784948), (4,0, 0,00448271794267), (5,0, 0,00542644803586), (6,0, 0,00637017812906), (7,0, 0,00731390822225), (8,0, 0,00825763831544), (9,0, 0,00731390822225), (10,0, 0,00637017812906), (11,0, 0,00542644803586), (12,0, 0,00448271794267), (13,0, 0,00353898784948), (14,0, 0,00259525775628), (15,0, 0,00165152766309), (16,0, 0,00165152766309), (17,0, 0,00165152766309), (18,0, 0,00165152766309), (19,0, 0,00165152766309), (20,0, 0,00165152766309), (21,0, 0,00165152766309), (22,0, 0,00165152766309), (23,0, 0,00235932523298), (24,0, 0,00306712280288), (25,0, 0,00377492037277), (26,0, 0,00448271794267), (27,0, 0,00519051551256), (28,0, 0,00589831308246), (29,0, 0,00660611065235), (30,0, 0,00754984074555), (31,0, 0,00849357083874), (32,0, 0,00943730093193), (33,0, 0,0103810310251), (34,0, 0,0113247611183), (35,0, 0,0122684912115), (36,0, 0,0132122213047), (37,0, 0,0155715465377), (38,0, 0,0179308717707), (39,0, 0,0202901970037), (40,0, 0,0226495222366), (41,0, 0,0250088474696), (42,0, 0,0273681727026), (43,0, 0,0297274979356), (44,0, 0,0349180134482), (45,0, 0,0401085289607), (46,0, 0,0452990444733), (47,0, 0,0504895599858), (48,0, 0,0556800754984), (49,0, 0,060870591011), (50,0, 0,0660611065235), (51,0, 0,0644095788604), (52,0, 0,0627580511974), (53,0, 0,0611065235343), (54,0, 0,0594549958712), (55,0, 0,0578034682081), (56,0, 0,056151940545), (57,0, 0,0545004128819), (58,0, 0,0670048366167), (59,0, 0,0795092603515), (60,0, 0,0920136840864), (61,0, 0,104518107821), (62,0, 0,117022531556), (63,0, 0,129526955291), (64,0, 0,142031379026), (65,0, 0,148637489678), (66,0, 0,15524360033), (67,0, 0,161849710983), (68,0, 0,168455821635), (69,0, 0,175061932287), (70,0, 0,18166804294), (71,0, 0,188274153592), (72,0, 0,204081632653), (73,0, 0,219889111714), (74,0, 0,235696590775), (75,0, 0,251504069836), (76,0, 0,267311548897), (77,0, 0,283119027958), (78,0, 0,298926507019), (79,0, 0,305768550195), (80,0, 0,31261059337), (81,0, 0,319452636546), (82,0, 0,326294679722), (83,0, 0,333136722897), (84,0, 0,339978766073), (85,0, 0,346820809249), (86,0, 0,360033030553), (87,0, 0,373245251858), (88,0, 0,386457473163), (89,0, 0,399669694467), (90,0, 0,412881915772), (91,0, 0,426094137077), (92,0, 0,439306358382), (93,0, 0,437654830718), (94,0, 0,436003303055), (95,0, 0,434351775392), (96,0, 0,432700247729), (97,0, 0,431048720066), (98,0, 0,429397192403), (99,0, 0,42774566474), (100,0, 0,424678541937), (101,0, 0,421611419134), (102,0, 0,418544296331), (103,0, 0,415477173528), (104,0, 0,412410050725), (105,0, 0,409342927923), (106,0, 0,40627580512), (107,0, 0,445440603987), (108,0, 0,484605402855), (109,0, 0,523770201722), (110,0, 0,56293500059), (111,0, 0,602099799457), (112,0, 0,641264598325), (113,0, 0,680429397192), (114,0, 0,752624749322), (115,0, 0,824820101451), (116,0, 0,89701545358), (117,0, 0,96921080571), (118,0, 1,04140615784), (119,0, 1,11360150997), (120,0, 1,1857968621), (121,0, 1,2273209862), (122,0, 1,2688451103), (123,0, 1,3103692344), (124,0, 1,3518933585), (125,0, 1,3934174826), (126,0, 1,4349416067), (127,0, 1,4764657308), (128,0, 1,48590303173), (129,0, 1,49534033266), (130,0, 1,5047776336), (131,0, 1,51421493453), (132,0, 1,52365223546), (133,0, 1,53308953639), (134,0, 1,54252683732), (135,0, 1,51138374425), (136,0, 1,48024065117), (137,0, 1,4490975581), (138,0, 1,41795446502), (139,0, 1,38681137195), (140,0, 1,35566827887), (141,0, 1,3245251858), (142,0, 1,29361802524), (143,0, 1,26271086469), (144,0, 1,23180370414), (145,0, 1,20089654359), (146,0, 1,16998938304), (147,0, 1,13908222248), (148,0, 1,10817506193), (149,0, 1,0855255397), (150,0, 1,06287601746), (151,0, 1,04022649522), (152,0, 1,01757697299), (153,0, 0,994927450749), (154,0, 0,972277928512), (155,0, 0,949628406276), (156,0, 0,927450749086), (157,0, 0,905273091896), (158,0, 0,883095434706), (159,0, 0,860917777516), (160,0, 0,838740120326), (161,0, 0,816562463136), (162,0, 0,794384805946), (163,0, 0,779756989501), (164,0, 0,765129173057), (165,0, 0,750501356612), (166,0, 0,735873540168), (167,0, 0,721245723723), (168,0, 0,706617907279), (169,0, 0,691990090834), (170,0, 0,685619912705), (171,0, 0,679249734576), (172,0, 0,672879556447), (173,0, 0,666509378318), (174,0, 0,660139200189), (175,0, 0,65376902206), (176,0, 0,647398843931), (177,0, 0,63229916244), (178,0, 0,617199480948), (179,0, 0,602099799457), (180,0, 0,587000117966), (181,0, 0,571900436475), (182,0, 0,556800754984), (183,0, 0,541701073493), (184,0, 0,515512563407), (185,0, 0,489324053321), (186,0, 0,463135543235), (187,0, 0,436947033149), (188,0, 0,410758523062), (189,0, 0,384570012976), (190,0, 0,35838150289), (191,0, 0,338091305887), (192,0, 0,317801108883), (193,0, 0,297510911879), (194,0, 0,277220714876), (195,0, 0,256930517872), (196,0, 0,236640320868), (197,0, 0,216350123865), (198,0, 0,205733160316), (199,0, 0,195116196768), (200,0, 0,184499233219), (201,0, 0,173882269671), (202,0, 0,163265306122), (203,0, 0,152648342574), (204,0, 0,142031379026), (205,0, 0,135661200897), (206,0, 0,129291022767), (207,0, 0,122920844638), (208,0, 0,116550666509), (209,0, 0,11018048838), (210,0, 0,103810310251), (211,0, 0,0974401321222), (212,0, 0,0972041995989), (213,0, 0,0969682670756), (214,0, 0,0967323345523), (215,0, 0,096496402029), (216,0, 0,0962604695057), (217,0, 0,0960245369824), (218,0, 0,0957886044591), (219,0, 0,0821045181078), (220,0, 0,0684204317565), (221,0, 0,0547363454052), (222,0, 0,0410522590539), (223,0, 0,0273681727026), (224,0, 0,0136840863513), (225,0, 0,000) |  |
| "Season_16--17" | GRAPH(TIME) Points: (1,0, 0,0148630880924), (2,0, 0,0138831042621), (3,0, 0,0129031204318), (4,0, 0,0119231366016), (5,0, 0,0109431527713), (6,0, 0,00996316894105), (7,0, 0,00898318511078), (8,0, 0,00800320128051), (9,0, 0,00718654808862), (10,0, 0,00636989489673), (11,0, 0,00555324170485), (12,0, 0,00473658851296), (13,0, 0,00391993532107), (14,0, 0,00310328212918), (15,0, 0,00228662893729), (16,0, 0,00261329021404), (17,0, 0,0029399514908), (18,0, 0,00326661276756), (19,0, 0,00359327404431), (20,0, 0,00391993532107), (21,0, 0,00424659659782), (22,0, 0,00457325787458), (23,0, 0,00473658851296), (24,0, 0,00489991915133), (25,0, 0,00506324978971), (26,0, 0,00522658042809), (27,0, 0,00538991106647), (28,0, 0,00555324170485), (29,0, 0,00571657234322), (30,0, 0,0112698140481), (31,0, 0,0168230557529), (32,0, 0,0223762974578), (33,0, 0,0279295391626), (34,0, 0,0334827808674), (35,0, 0,0390360225723), (36,0, 0,0445892642771), (37,0, 0,0473658851296), (38,0, 0,050142505982), (39,0, 0,0529191268344), (40,0, 0,0556957476868), (41,0, 0,0584723685393), (42,0, 0,0612489893917), (43,0, 0,0640256102441), (44,0, 0,0712121583327), (45,0, 0,0783987064213), (46,0, 0,08558525451), (47,0, 0,0927718025986), (48,0, 0,0999583506872), (49,0, 0,107144898776), (50,0, 0,114331446864), (51,0, 0,122334648145), (52,0, 0,130337849425), (53,0, 0,138341050706), (54,0, 0,146344251987), (55,0, 0,154347453267), (56,0, 0,162350654548), (57,0, 0,170353855828), (58,0, 0,200896685205), (59,0, 0,231439514581), (60,0, 0,261982343958), (61,0, 0,292525173335), (62,0, 0,323068002711), (63,0, 0,353610832088), (64,0, 0,384153661465), (65,0, 0,414206498926), (66,0, 0,444259336388), (67,0, 0,474312173849), (68,0, 0,504365011311), (69,0, 0,534417848772), (70,0, 0,564470686234), (71,0, 0,594523523695), (72,0, 0,678312141183), (73,0, 0,762100758671), (74,0, 0,845889376159), (75,0, 0,929677993646), (76,0, 1,01346661113), (77,0, 1,09725522862), (78,0, 1,18104384611), (79,0, 1,23102302145), (80,0, 1,2810021968), (81,0, 1,33098137214), (82,0, 1,38096054748), (83,0, 1,43093972283), (84,0, 1,48091889817), (85,0, 1,53089807352), (86,0, 1,46213587476), (87,0, 1,393373676), (88,0, 1,32461147724), (89,0, 1,25584927849), (90,0, 1,18708707973), (91,0, 1,11832488097), (92,0, 1,04956268222), (93,0, 1,01983650603), (94,0, 0,990110329846), (95,0, 0,960384153661), (96,0, 0,930657977477), (97,0, 0,900931801292), (98,0, 0,871205625107), (99,0, 0,841479448922), (100,0, 0,818776490188), (101,0, 0,796073531453), (102,0, 0,773370572719), (103,0, 0,750667613984), (104,0, 0,72796465525), (105,0, 0,705261696515), (106,0, 0,682558737781), (107,0, 0,690235277785), (108,0, 0,697911817788), (109,0, 0,705588357792), (110,0, 0,713264897796), (111,0, 0,7209414378), (112,0, 0,728617977803), (113,0, 0,736294517807), (114,0, 0,752790912283), (115,0, 0,769287306759), (116,0, 0,785783701236), (117,0, 0,802280095712), (118,0, 0,818776490188), (119,0, 0,835272884664), (120,0, 0,85176927914), (121,0, 0,866469036594), (122,0, 0,881168794048), (123,0, 0,895868551502), (124,0, 0,910568308956), (125,0, 0,92526806641), (126,0, 0,939967823864), (127,0, 0,954667581318), (128,0, 0,962017460045), (129,0, 0,969367338772), (130,0, 0,976717217499), (131,0, 0,984067096226), (132,0, 0,991416974953), (133,0, 0,99876685368), (134,0, 1,00611673241), (135,0, 1,01232329667), (136,0, 1,01852986092), (137,0, 1,02473642518), (138,0, 1,03094298944), (139,0, 1,0371495537), (140,0, 1,04335611796), (141,0, 1,04956268222), (142,0, 1,00807670007), (143,0, 0,96659071792), (144,0, 0,925104735772), (145,0, 0,883618753624), (146,0, 0,842132771476), (147,0, 0,800646789328), (148,0, 0,75916080718), (149,0, 0,722574744183), (150,0, 0,685988681187), (151,0, 0,64940261819), (152,0, 0,612816555194), (153,0, 0,576230492197), (154,0, 0,5396444292), (155,0, 0,503058366204), (156,0, 0,492115213432), (157,0, 0,481172060661), (158,0, 0,47022890789), (159,0, 0,459285755118), (160,0, 0,448342602347), (161,0, 0,437399449576), (162,0, 0,426456296804), (163,0, 0,412083200627), (164,0, 0,39771010445), (165,0, 0,383337008273), (166,0, 0,368963912095), (167,0, 0,354590815918), (168,0, 0,340217719741), (169,0, 0,325844623564), (170,0, 0,31718809973), (171,0, 0,308531575896), (172,0, 0,299875052062), (173,0, 0,291218528228), (174,0, 0,282562004394), (175,0, 0,27390548056), (176,0, 0,265248956726), (177,0, 0,260512368213), (178,0, 0,2557757797), (179,0, 0,251039191187), (180,0, 0,246302602674), (181,0, 0,241566014161), (182,0, 0,236829425648), (183,0, 0,232092837135), (184,0, 0,220333031172), (185,0, 0,208573225208), (186,0, 0,196813419245), (187,0, 0,185053613282), (188,0, 0,173293807319), (189,0, 0,161534001356), (190,0, 0,149774195392), (191,0, 0,145200937518), (192,0, 0,140627679643), (193,0, 0,136054421769), (194,0, 0,131481163894), (195,0, 0,12690790602), (196,0, 0,122334648145), (197,0, 0,11776139027), (198,0, 0,116618075802), (199,0, 0,115474761333), (200,0, 0,114331446864), (201,0, 0,113188132396), (202,0, 0,112044817927), (203,0, 0,110901503459), (204,0, 0,10975818899), (205,0, 0,109104866436), (206,0, 0,108451543883), (207,0, 0,107798221329), (208,0, 0,107144898776), (209,0, 0,106491576222), (210,0, 0,105838253669), (211,0, 0,105184931115), (212,0, 0,10567492303), (213,0, 0,106164914946), (214,0, 0,106654906861), (215,0, 0,107144898776), (216,0, 0,107634890691), (217,0, 0,108124882606), (218,0, 0,108614874521), (219,0, 0,109268197075), (220,0, 0,109921519628), (221,0, 0,110574842182), (222,0, 0,111228164735), (223,0, 0,111881487289), (224,0, 0,112534809842), (225,0, 0,113188132396) |  |
| "Season_17--18" | GRAPH(TIME) Points: (1,0, 0,00697987701457), (2,0, 0,00707958954335), (3,0, 0,00717930207213), (4,0, 0,00727901460091), (5,0, 0,00737872712969), (6,0, 0,00747843965846), (7,0, 0,00757815218724), (8,0, 0,00767786471602), (9,0, 0,00697987701457), (10,0, 0,00628188931311), (11,0, 0,00558390161165), (12,0, 0,0048859139102), (13,0, 0,00418792620874), (14,0, 0,00348993850728), (15,0, 0,00279195080583), (16,0, 0,00329051344972), (17,0, 0,00378907609362), (18,0, 0,00428763873752), (19,0, 0,00478620138142), (20,0, 0,00528476402532), (21,0, 0,00578332666921), (22,0, 0,00628188931311), (23,0, 0,00648131437067), (24,0, 0,00668073942823), (25,0, 0,00688016448579), (26,0, 0,00707958954335), (27,0, 0,00727901460091), (28,0, 0,00747843965846), (29,0, 0,00767786471602), (30,0, 0,00907384011894), (31,0, 0,0104698155219), (32,0, 0,0118657909248), (33,0, 0,0132617663277), (34,0, 0,0146577417306), (35,0, 0,0160537171335), (36,0, 0,0174496925364), (37,0, 0,0197430806983), (38,0, 0,0220364688603), (39,0, 0,0243298570222), (40,0, 0,0266232451841), (41,0, 0,0289166333461), (42,0, 0,031210021508), (43,0, 0,0335034096699), (44,0, 0,0354976602455), (45,0, 0,0374919108211), (46,0, 0,0394861613967), (47,0, 0,0414804119723), (48,0, 0,0434746625479), (49,0, 0,0454689131235), (50,0, 0,0474631636991), (51,0, 0,0472637386415), (52,0, 0,0470643135839), (53,0, 0,0468648885264), (54,0, 0,0466654634688), (55,0, 0,0464660384113), (56,0, 0,0462666133537), (57,0, 0,0460671882961), (58,0, 0,0512522397927), (59,0, 0,0564372912892), (60,0, 0,0616223427857), (61,0, 0,0668073942823), (62,0, 0,0719924457788), (63,0, 0,0771774972754), (64,0, 0,0823625487719), (65,0, 0,0890432882001), (66,0, 0,0957240276283), (67,0, 0,102404767057), (68,0, 0,109085506485), (69,0, 0,115766245913), (70,0, 0,122446985341), (71,0, 0,129127724769), (72,0, 0,144084604086), (73,0, 0,159041483403), (74,0, 0,17399836272), (75,0, 0,188955242037), (76,0, 0,203912121354), (77,0, 0,218869000671), (78,0, 0,233825879988), (79,0, 0,251474997582), (80,0, 0,269124115176), (81,0, 0,28677323277), (82,0, 0,304422350364), (83,0, 0,322071467958), (84,0, 0,339720585552), (85,0, 0,357369703146), (86,0, 0,370033194301), (87,0, 0,382696685456), (88,0, 0,395360176611), (89,0, 0,408023667766), (90,0, 0,420687158921), (91,0, 0,433350650076), (92,0, 0,446014141231), (93,0, 0,444119603184), (94,0, 0,442225065137), (95,0, 0,44033052709), (96,0, 0,438435989044), (97,0, 0,436541450997), (98,0, 0,43464691295), (99,0, 0,432752374903), (100,0, 0,443122477896), (101,0, 0,453492580889), (102,0, 0,463862683882), (103,0, 0,474232786875), (104,0, 0,484602889869), (105,0, 0,494972992862), (106,0, 0,505343095855), (107,0, 0,541439031273), (108,0, 0,577534966691), (109,0, 0,613630902109), (110,0, 0,649726837527), (111,0, 0,685822772946), (112,0, 0,721918708364), (113,0, 0,758014643782), (114,0, 0,795506554603), (115,0, 0,832998465424), (116,0, 0,870490376245), (117,0, 0,907982287066), (118,0, 0,945474197887), (119,0, 0,982966108709), (120,0, 1,02045801953), (121,0, 1,06104101874), (122,0, 1,10162401796), (123,0, 1,14220701717), (124,0, 1,18279001638), (125,0, 1,2233730156), (126,0, 1,26395601481), (127,0, 1,30453901402), (128,0, 1,33335593484), (129,0, 1,36217285566), (130,0, 1,39098977647), (131,0, 1,41980669729), (132,0, 1,44862361811), (133,0, 1,47744053893), (134,0, 1,50625745974), (135,0, 1,48471955353), (136,0, 1,46318164731), (137,0, 1,44164374109), (138,0, 1,42010583488), (139,0, 1,39856792866), (140,0, 1,37703002245), (141,0, 1,35549211623), (142,0, 1,33594846059), (143,0, 1,31640480495), (144,0, 1,29686114931), (145,0, 1,27731749367), (146,0, 1,25777383802), (147,0, 1,23823018238), (148,0, 1,21868652674), (149,0, 1,20243338455), (150,0, 1,18618024236), (151,0, 1,16992710017), (152,0, 1,15367395798), (153,0, 1,13742081579), (154,0, 1,1211676736), (155,0, 1,10491453141), (156,0, 1,0723085345), (157,0, 1,03970253758), (158,0, 1,00709654067), (159,0, 0,974490543762), (160,0, 0,941884546851), (161,0, 0,909278549941), (162,0, 0,87667255303), (163,0, 0,868894975785), (164,0, 0,86111739854), (165,0, 0,853339821295), (166,0, 0,84556224405), (167,0, 0,837784666806), (168,0, 0,830007089561), (169,0, 0,822229512316), (170,0, 0,794509429315), (171,0, 0,766789346315), (172,0, 0,739069263314), (173,0, 0,711349180313), (174,0, 0,683629097312), (175,0, 0,655909014312), (176,0, 0,628188931311), (177,0, 0,617918540847), (178,0, 0,607648150382), (179,0, 0,597377759918), (180,0, 0,587107369454), (181,0, 0,57683697899), (182,0, 0,566566588525), (183,0, 0,556296198061), (184,0, 0,525385314139), (185,0, 0,494474430218), (186,0, 0,463563546296), (187,0, 0,432652662374), (188,0, 0,401741778453), (189,0, 0,370830894531), (190,0, 0,339920010609), (191,0, 0,324065718533), (192,0, 0,308211426458), (193,0, 0,292357134382), (194,0, 0,276502842306), (195,0, 0,26064855023), (196,0, 0,244794258154), (197,0, 0,228939966078), (198,0, 0,211091423426), (199,0, 0,193242880775), (200,0, 0,175394338123), (201,0, 0,157545795472), (202,0, 0,13969725282), (203,0, 0,121848710169), (204,0, 0,104000167517), (205,0, 0,0977182782039), (206,0, 0,0914363888908), (207,0, 0,0851544995777), (208,0, 0,0788726102646), (209,0, 0,0725907209515), (210,0, 0,0663088316384), (211,0, 0,0600269423253), (212,0, 0,0569358539331), (213,0, 0,0538447655409), (214,0, 0,0507536771488), (215,0, 0,0476625887566), (216,0, 0,0445715003644), (217,0, 0,0414804119723), (218,0, 0,0383893235801), (219,0, 0,0329051344972), (220,0, 0,0274209454144), (221,0, 0,0219367563315), (222,0, 0,0164525672486), (223,0, 0,0109683781657), (224,0, 0,00548418908287), (225,0, 0,000) |  |
| "Season_18--19" | GRAPH(TIME) Points: (1,0, 0,00529296564865), (2,0, 0,00559542082858), (3,0, 0,0058978760085), (4,0, 0,00620033118842), (5,0, 0,00650278636835), (6,0, 0,00680524154827), (7,0, 0,00710769672819), (8,0, 0,00741015190811), (9,0, 0,00756137949808), (10,0, 0,00771260708804), (11,0, 0,007863834678), (12,0, 0,00801506226796), (13,0, 0,00816628985792), (14,0, 0,00831751744788), (15,0, 0,00846874503784), (16,0, 0,00801506226796), (17,0, 0,00756137949808), (18,0, 0,00710769672819), (19,0, 0,00665401395831), (20,0, 0,00620033118842), (21,0, 0,00574664841854), (22,0, 0,00529296564865), (23,0, 0,00529296564865), (24,0, 0,00529296564865), (25,0, 0,00529296564865), (26,0, 0,00529296564865), (27,0, 0,00529296564865), (28,0, 0,00529296564865), (29,0, 0,00529296564865), (30,0, 0,00635155877838), (31,0, 0,00741015190811), (32,0, 0,00846874503784), (33,0, 0,00952733816758), (34,0, 0,0105859312973), (35,0, 0,011644524427), (36,0, 0,0127031175568), (37,0, 0,0133080279166), (38,0, 0,0139129382765), (39,0, 0,0145178486363), (40,0, 0,0151227589962), (41,0, 0,015727669356), (42,0, 0,0163325797158), (43,0, 0,0169374900757), (44,0, 0,0172399452556), (45,0, 0,0175424004355), (46,0, 0,0178448556155), (47,0, 0,0181473107954), (48,0, 0,0184497659753), (49,0, 0,0187522211552), (50,0, 0,0190546763352), (51,0, 0,0238939592139), (52,0, 0,0287332420927), (53,0, 0,0335725249715), (54,0, 0,0384118078502), (55,0, 0,043251090729), (56,0, 0,0480903736078), (57,0, 0,0529296564865), (58,0, 0,0615496291143), (59,0, 0,0701696017421), (60,0, 0,0787895743699), (61,0, 0,0874095469978), (62,0, 0,0960295196256), (63,0, 0,104649492253), (64,0, 0,113269464881), (65,0, 0,129299589417), (66,0, 0,145329713953), (67,0, 0,161359838489), (68,0, 0,177389963025), (69,0, 0,193420087561), (70,0, 0,209450212097), (71,0, 0,225480336633), (72,0, 0,256330764985), (73,0, 0,287181193337), (74,0, 0,318031621689), (75,0, 0,348882050041), (76,0, 0,379732478393), (77,0, 0,410582906746), (78,0, 0,441433335098), (79,0, 0,494514219174), (80,0, 0,547595103251), (81,0, 0,600675987327), (82,0, 0,653756871404), (83,0, 0,70683775548), (84,0, 0,759918639557), (85,0, 0,812999523633), (86,0, 0,817233896152), (87,0, 0,821468268671), (88,0, 0,82570264119), (89,0, 0,829937013709), (90,0, 0,834171386228), (91,0, 0,838405758747), (92,0, 0,842640131266), (93,0, 0,830390696479), (94,0, 0,818141261692), (95,0, 0,805891826905), (96,0, 0,793642392118), (97,0, 0,781392957331), (98,0, 0,769143522544), (99,0, 0,756894087757), (100,0, 0,755381811858), (101,0, 0,753869535958), (102,0, 0,752357260059), (103,0, 0,750844984159), (104,0, 0,749332708259), (105,0, 0,74782043236), (106,0, 0,74630815646), (107,0, 0,773680350243), (108,0, 0,801052544026), (109,0, 0,828424737809), (110,0, 0,855796931592), (111,0, 0,883169125375), (112,0, 0,910541319158), (113,0, 0,937913512941), (114,0, 0,98131583126), (115,0, 1,02471814958), (116,0, 1,0681204679), (117,0, 1,11152278622), (118,0, 1,15492510454), (119,0, 1,19832742286), (120,0, 1,24172974117), (121,0, 1,26017950715), (122,0, 1,27862927312), (123,0, 1,2970790391), (124,0, 1,31552880508), (125,0, 1,33397857105), (126,0, 1,35242833703), (127,0, 1,370878103), (128,0, 1,35696516472), (129,0, 1,34305222645), (130,0, 1,32913928817), (131,0, 1,3152263499), (132,0, 1,30131341162), (133,0, 1,28740047334), (134,0, 1,27348753507), (135,0, 1,23643677553), (136,0, 1,19938601598), (137,0, 1,16233525644), (138,0, 1,1252844969), (139,0, 1,08823373736), (140,0, 1,05118297782), (141,0, 1,01413221828), (142,0, 0,979803555361), (143,0, 0,945474892439), (144,0, 0,911146229518), (145,0, 0,876817566597), (146,0, 0,842488903676), (147,0, 0,808160240754), (148,0, 0,773831577833), (149,0, 0,749332708259), (150,0, 0,724833838686), (151,0, 0,700334969112), (152,0, 0,675836099538), (153,0, 0,651337229964), (154,0, 0,62683836039), (155,0, 0,602339490817), (156,0, 0,593719518189), (157,0, 0,585099545561), (158,0, 0,576479572933), (159,0, 0,567859600305), (160,0, 0,559239627678), (161,0, 0,55061965505), (162,0, 0,541999682422), (163,0, 0,533077254614), (164,0, 0,524154826807), (165,0, 0,515232398999), (166,0, 0,506309971191), (167,0, 0,497387543383), (168,0, 0,488465115576), (169,0, 0,479542687768), (170,0, 0,46986412201), (171,0, 0,460185556253), (172,0, 0,450506990495), (173,0, 0,440828424738), (174,0, 0,43114985898), (175,0, 0,421471293223), (176,0, 0,411792727465), (177,0, 0,407709582536), (178,0, 0,403626437607), (179,0, 0,399543292678), (180,0, 0,395460147749), (181,0, 0,39137700282), (182,0, 0,387293857891), (183,0, 0,383210712962), (184,0, 0,384118078502), (185,0, 0,385025444042), (186,0, 0,385932809582), (187,0, 0,386840175122), (188,0, 0,387747540661), (189,0, 0,388654906201), (190,0, 0,389562271741), (191,0, 0,379278795623), (192,0, 0,368995319506), (193,0, 0,358711843389), (194,0, 0,348428367271), (195,0, 0,338144891154), (196,0, 0,327861415037), (197,0, 0,317577938919), (198,0, 0,317426711329), (199,0, 0,317275483739), (200,0, 0,317124256149), (201,0, 0,316973028559), (202,0, 0,316821800969), (203,0, 0,316670573379), (204,0, 0,316519345789), (205,0, 0,293986434885), (206,0, 0,271453523981), (207,0, 0,248920613077), (208,0, 0,226387702172), (209,0, 0,203854791268), (210,0, 0,181321880364), (211,0, 0,15878896946), (212,0, 0,136104830965), (213,0, 0,113420692471), (214,0, 0,0907365539769), (215,0, 0,0680524154827), (216,0, 0,0453682769885), (217,0, 0,0226841384942), (218,0, 0,000), (219,0, 0,000), (220,0, 0,000), (221,0, 0,000), (222,0, 0,000), (223,0, 0,000), (224,0, 0,000), (225,0, 0,000) |  |
| Season_free | GRAPH(TIME) Points: (1,0, 0,011261895377), (2,0, 0,0104574742786), (3,0, 0,00965305318028), (4,0, 0,00884863208192), (5,0, 0,00804421098357), (6,0, 0,00723978988521), (7,0, 0,00643536878685), (8,0, 0,0056309476885), (9,0, 0,0056309476885), (10,0, 0,0056309476885), (11,0, 0,0056309476885), (12,0, 0,0056309476885), (13,0, 0,0056309476885), (14,0, 0,0056309476885), (15,0, 0,0056309476885), (16,0, 0,00643536878685), (17,0, 0,00723978988521), (18,0, 0,00804421098357), (19,0, 0,00884863208192), (20,0, 0,00965305318028), (21,0, 0,0104574742786), (22,0, 0,011261895377), (23,0, 0,0144795797704), (24,0, 0,0176972641638), (25,0, 0,0209149485573), (26,0, 0,0241326329507), (27,0, 0,0273503173441), (28,0, 0,0305680017375), (29,0, 0,033785686131), (30,0, 0,0329812650326), (31,0, 0,0321768439343), (32,0, 0,0313724228359), (33,0, 0,0305680017375), (34,0, 0,0297635806392), (35,0, 0,0289591595408), (36,0, 0,0281547384425), (37,0, 0,0313724228359), (38,0, 0,0345901072293), (39,0, 0,0378077916228), (40,0, 0,0410254760162), (41,0, 0,0442431604096), (42,0, 0,047460844803), (43,0, 0,0506785291965), (44,0, 0,0458520026063), (45,0, 0,0410254760162), (46,0, 0,036198949426), (47,0, 0,0313724228359), (48,0, 0,0265458962458), (49,0, 0,0217193696556), (50,0, 0,0168928430655), (51,0, 0,0193061063606), (52,0, 0,0217193696556), (53,0, 0,0241326329507), (54,0, 0,0265458962458), (55,0, 0,0289591595408), (56,0, 0,0313724228359), (57,0, 0,033785686131), (58,0, 0,0402210549178), (59,0, 0,0466564237047), (60,0, 0,0530917924915), (61,0, 0,0595271612784), (62,0, 0,0659625300652), (63,0, 0,0723978988521), (64,0, 0,0788332676389), (65,0, 0,0868774786225), (66,0, 0,0949216896061), (67,0, 0,10296590059), (68,0, 0,111010111573), (69,0, 0,119054322557), (70,0, 0,12709853354), (71,0, 0,135142744524), (72,0, 0,177777062737), (73,0, 0,22041138095), (74,0, 0,263045699163), (75,0, 0,305680017375), (76,0, 0,348314335588), (77,0, 0,390948653801), (78,0, 0,433582972014), (79,0, 0,440018340801), (80,0, 0,446453709588), (81,0, 0,452889078375), (82,0, 0,459324447162), (83,0, 0,465759815948), (84,0, 0,472195184735), (85,0, 0,478630553522), (86,0, 0,483457080112), (87,0, 0,488283606702), (88,0, 0,493110133293), (89,0, 0,497936659883), (90,0, 0,502763186473), (91,0, 0,507589713063), (92,0, 0,512416239653), (93,0, 0,516438345145), (94,0, 0,520460450637), (95,0, 0,524482556128), (96,0, 0,52850466162), (97,0, 0,532526767112), (98,0, 0,536548872604), (99,0, 0,540570978096), (100,0, 0,518047187342), (101,0, 0,495523396588), (102,0, 0,472999605834), (103,0, 0,45047581508), (104,0, 0,427952024326), (105,0, 0,405428233572), (106,0, 0,382904442818), (107,0, 0,402210549178), (108,0, 0,421516655539), (109,0, 0,440822761899), (110,0, 0,46012886826), (111,0, 0,479434974621), (112,0, 0,498741080981), (113,0, 0,518047187342), (114,0, 0,551028452374), (115,0, 0,584009717407), (116,0, 0,616990982439), (117,0, 0,649972247472), (118,0, 0,682953512505), (119,0, 0,715934777537), (120,0, 0,74891604257), (121,0, 0,820509520324), (122,0, 0,892102998077), (123,0, 0,963696475831), (124,0, 1,03528995358), (125,0, 1,10688343134), (126,0, 1,17847690909), (127,0, 1,25007038685), (128,0, 1,28868259957), (129,0, 1,32729481229), (130,0, 1,36590702501), (131,0, 1,40451923773), (132,0, 1,44313145045), (133,0, 1,48174366317), (134,0, 1,52035587589), (135,0, 1,55172829873), (136,0, 1,58310072157), (137,0, 1,6144731444), (138,0, 1,64584556724), (139,0, 1,67721799007), (140,0, 1,70859041291), (141,0, 1,73996283575), (142,0, 1,67802241117), (143,0, 1,6160819866), (144,0, 1,55414156202), (145,0, 1,49220113745), (146,0, 1,43026071288), (147,0, 1,3683202883), (148,0, 1,30637986373), (149,0, 1,2742030198), (150,0, 1,24202617586), (151,0, 1,20984933193), (152,0, 1,17767248799), (153,0, 1,14549564406), (154,0, 1,11331880013), (155,0, 1,08114195619), (156,0, 1,04735627006), (157,0, 1,01357058393), (158,0, 0,979784897798), (159,0, 0,945999211667), (160,0, 0,912213525536), (161,0, 0,878427839405), (162,0, 0,844642153274), (163,0, 0,808443203848), (164,0, 0,772244254422), (165,0, 0,736045304996), (166,0, 0,69984635557), (167,0, 0,663647406144), (168,0, 0,627448456718), (169,0, 0,591249507292), (170,0, 0,575965506423), (171,0, 0,560681505555), (172,0, 0,545397504686), (173,0, 0,530113503817), (174,0, 0,514829502948), (175,0, 0,499545502079), (176,0, 0,484261501211), (177,0, 0,471390763637), (178,0, 0,458520026063), (179,0, 0,44564928849), (180,0, 0,432778550916), (181,0, 0,419907813342), (182,0, 0,407037075768), (183,0, 0,394166338195), (184,0, 0,398992864785), (185,0, 0,403819391375), (186,0, 0,408645917965), (187,0, 0,413472444555), (188,0, 0,418298971145), (189,0, 0,423125497736), (190,0, 0,427952024326), (191,0, 0,403819391375), (192,0, 0,379686758424), (193,0, 0,355554125474), (194,0, 0,331421492523), (195,0, 0,307288859572), (196,0, 0,283156226622), (197,0, 0,259023593671), (198,0, 0,259023593671), (199,0, 0,259023593671), (200,0, 0,259023593671), (201,0, 0,259023593671), (202,0, 0,259023593671), (203,0, 0,259023593671), (204,0, 0,259023593671), (205,0, 0,236499802917), (206,0, 0,213976012163), (207,0, 0,191452221409), (208,0, 0,168928430655), (209,0, 0,146404639901), (210,0, 0,123880849147), (211,0, 0,101357058393), (212,0, 0,0868774786225), (213,0, 0,0723978988521), (214,0, 0,0579183190817), (215,0, 0,0434387393113), (216,0, 0,0289591595408), (217,0, 0,0144795797704), (218,0, 0,000), (219,0, 0,000), (220,0, 0,000), (221,0, 0,000), (222,0, 0,000), (223,0, 0,000), (224,0, 0,000), (225,0, 0,000) |  |
| "Share_of_HCAI_treated_day_2_(%)" | 60 |  |
| "Share_of_INS_treated_day_2_(%)" | 5 |  |
| "Share_of_IS_treated_day_2_(%)" | 40 |  |
| Total_#_influenza_patients_from_Emergency | 500 |  |
| Total_ED_arrivals_per_year | 15000 |  |
| "Vaccination_coverage_(%)" | 50 |  |
| "Vaccine_effectiveness_(%)" | 75 |  |
| "Viral_titre_level_day_1_(treated)" | ,432 |  |
| "Viral_titre_level_day_1_(untreated)" | ,432 |  |
| "Viral_titre_level_day_2_(treated)" | 1,486 |  |
| "Viral_titre_level_day_2_(untreated)" | 1,486 |  |
| "Viral_titre_level_day_3_(treated)" | 2,351 |  |
| "Viral_titre_level_day_3_(untreated)" | 2,351 |  |
| "Viral_titre_level_day_4_(treated)" | 1,027 |  |
| "Viral_titre_level_day_4_(untreated)" | 1,730 |  |
| "Viral_titre_level_day_5_(treated)" | ,541 |  |
| "Viral_titre_level_day_5_(untreated)" | 1,027 |  |
| "Viral_titre_level_day_6_(treated)" | ,297 |  |
| "Viral_titre_level_day_6_(untreated)" | 0,541 |  |
| "Viral_titre_level_day_7_(treated)" | 0,135 |  |
| "Viral_titre_level_day_7_(untreated)" | 0,297 |  |
| "Viral_titre_level_day_8_(treated)" | 0,0001 |  |
| "Viral_titre_level_day_8_(untreated)" | 0,135 |  |

| Total | Count | Including Array Elements |
| --- | --- | --- |
| Variables | 140 | 140 |
| Stocks | 33 | 33 |
| Flows | 34 | 34 |
| Converters | 73 | 73 |
| Constants | 57 | 57 |
| Equations | 50 | 50 |
| Graphicals | 7 | 7 |

| Run Specs | |
| --- | --- |
| Start Time | 1 |
| Stop Time | 225 |
| DT | 1/4 |
| Fractional DT | True |
| Save Interval | 0,25 |
| Sim Duration | 1,49333408 |
| Time Units | Days |
| Pause Interval | 0 |
| Integration Method | Euler |
| Keep all variable results | True |
| Run By | Run |
| Calculate loop dominance information | False |
