# Supplementary material for: System dynamic modelling of healthcare associated influenza -a tool for infection control
Source: BMC Health Serv Res. 2022 May 27;22:709. doi: 10.1186/s12913-022-07959-7 (PMC9136787; doi:10.1186/s12913-022-07959-7)

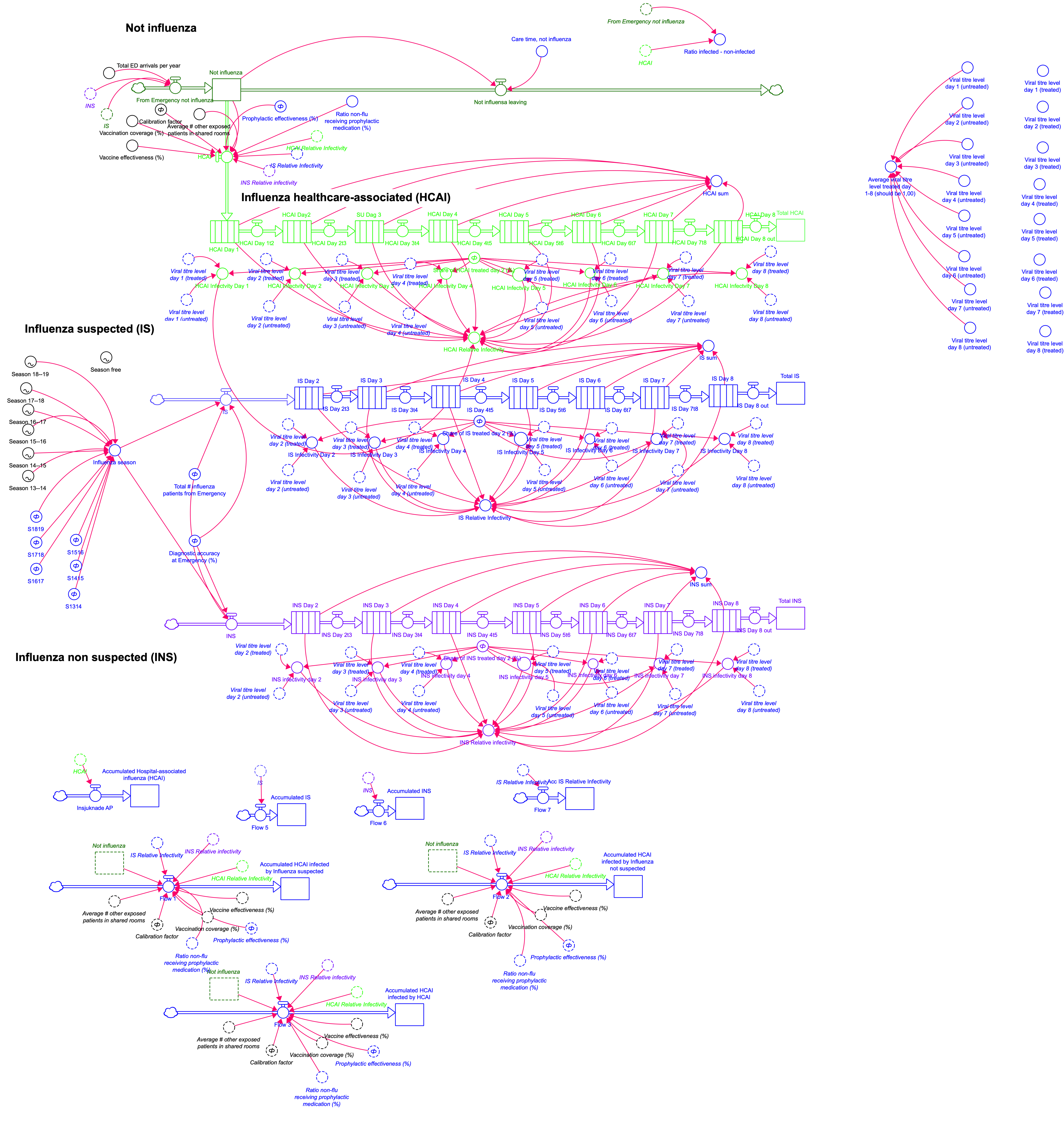

BY

This model is a companion to the paper
System dynamic modelling of healthcare-associated influenza - a tool for infection control
by
M. Sansone, P. Holmström, S. Hallberg, R. Nordén, L-M Andersson, J. Westin

Exposure

Average # other exposed patients in shared rooms

0 1 2 3

Susceptibility

Vaccination coverage (%)

0 10 20 30 40 50 60 70 80 90 100

Vaccine effectiveness (%)

0 10 20 30 40 50 60 70 80 90 100

Ratio non-flu receiving prophylactic medication (%)

0 10 20 30 40 50 60 70 80 90 100

Prophylactic effectiveness (%)

0 10 20 30 40 50 60 70 80 90 100

Actions

Share of IS treated day 2 (%)

0 10 20 30 40 50 60 70 80 90 100

Share of INS treated day 2 (%)

0 10 20 30 40 50 60 70 80 90 100

Share of HCAI treated day 2 (%)

0 10 20 30 40 50 60 70 80 90 100

Diagnostic accuracy at Emergency (%)

0 10 20 30 40 50 60 70 80 90 100

BY

This model is a companion to the paper
System dynamic modelling of healthcare-associated influenza - a tool for infection control
by
M. Sansone, P. Holmström, S. Hallberg, R. Nordén, L-M Andersson, J. Westin

Accumulated Hospital-associated influenza (HCAI)	111
Accumulated HCAI infected by Influenza suspected	33
Accumulated HCAI infected by Influenza not suspected	57
Accumulated HCAI infected by HCAI	22

Calibration factor

0 0,2 0,4 0,6 0,8 1

Variables

Total # influenza patients from Emergency

0 500 1k 1,5k 2k

Season selector

- Season 2013-14
- Season 2014-15
- Season 2015-16
- Season 2016-17 (selected)
- Season 2017-18
- Season 2018-19

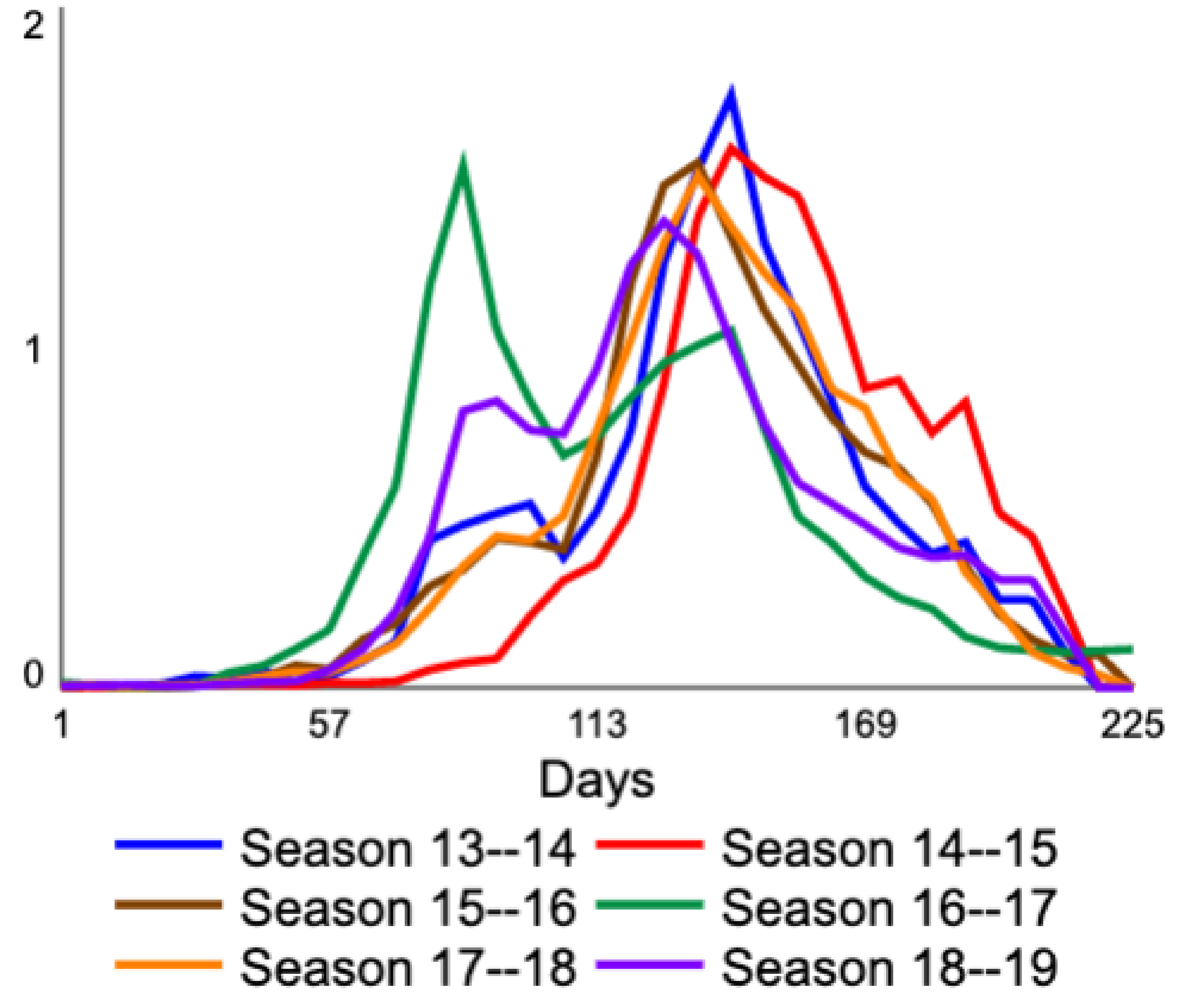

Run Live (real time)

Single run

Stop Live

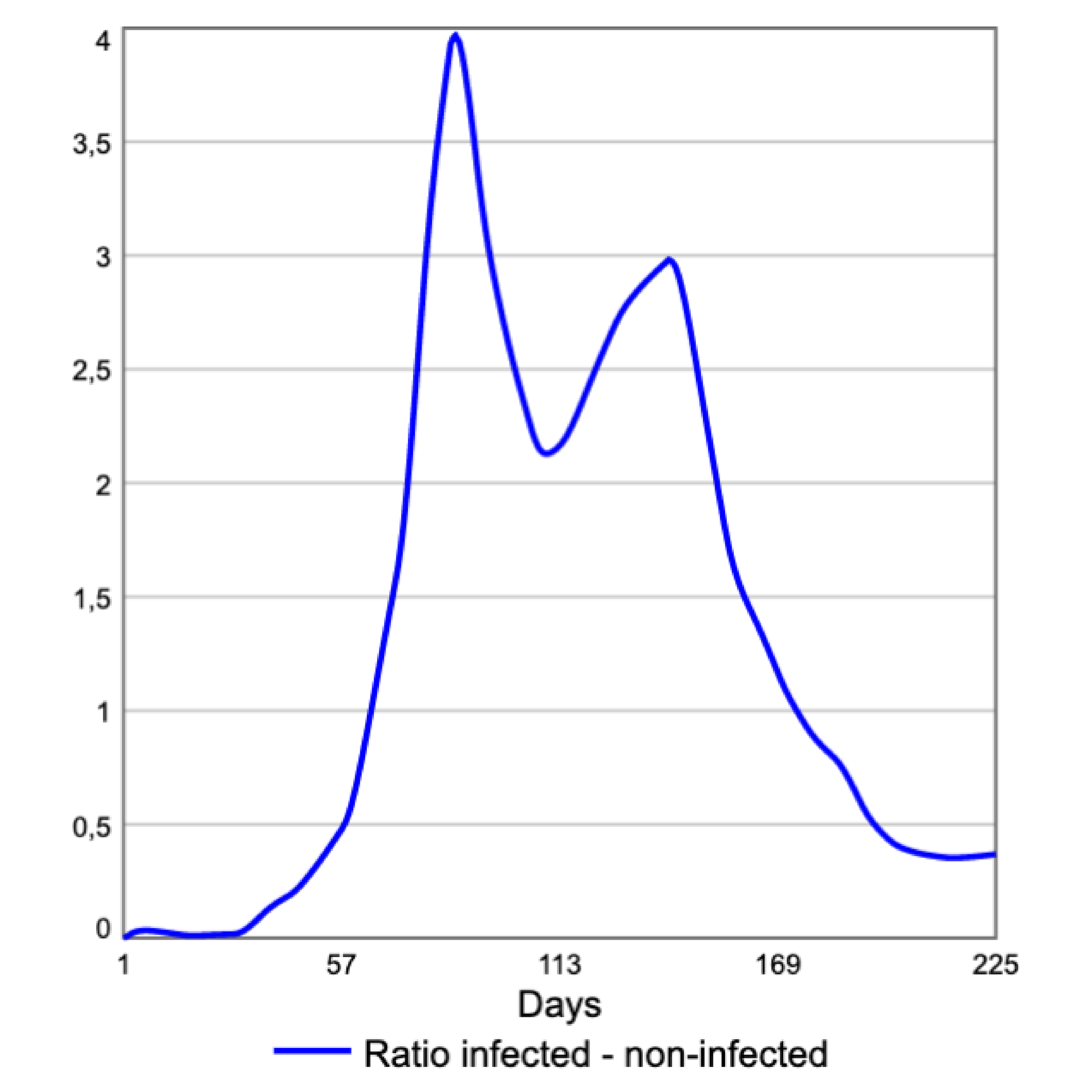

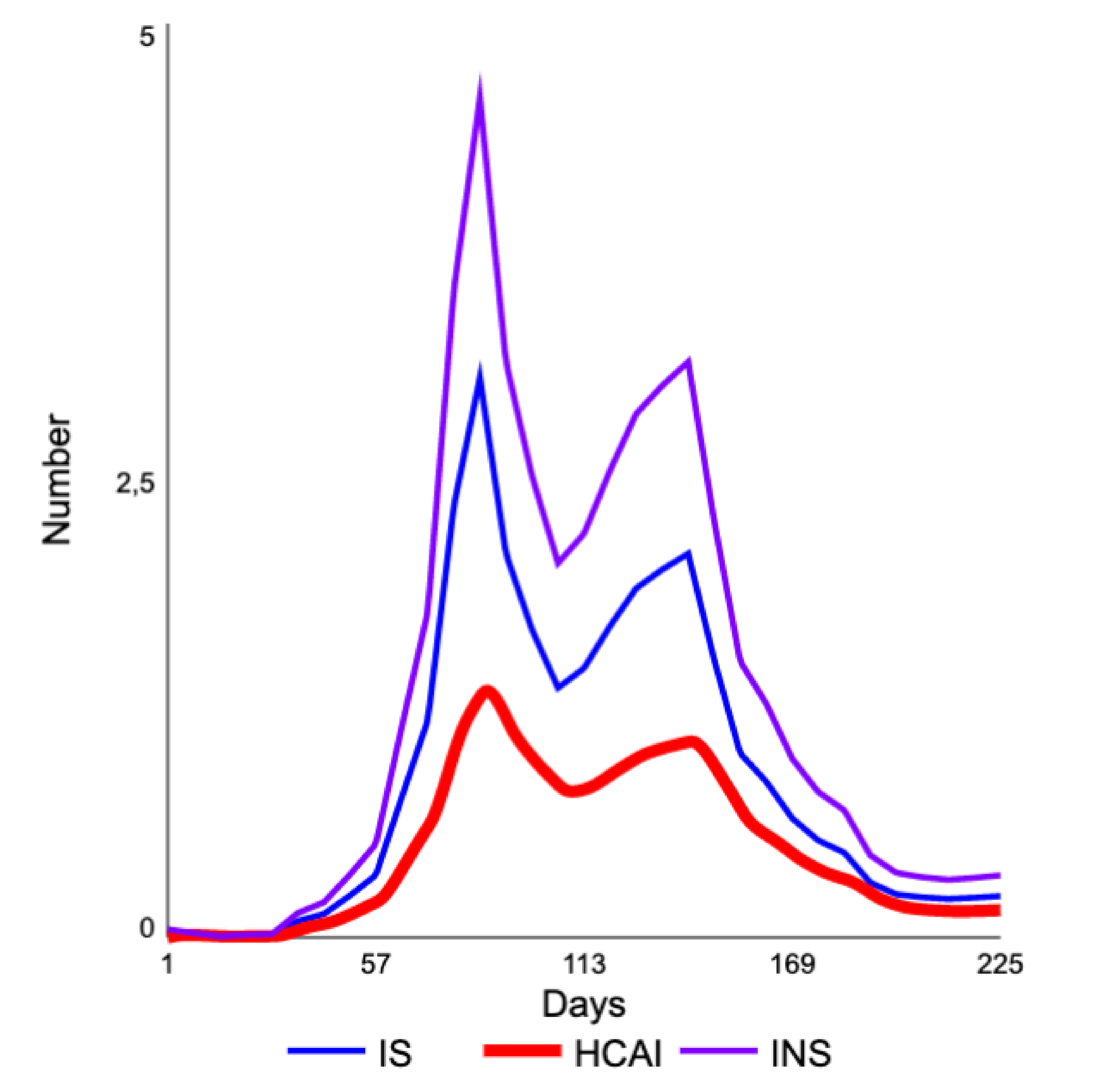

Supplement: Supplementary file 2 — Additional file 2. Detailed stock-and-flow diagram with user interface. [file 12913_2022_7959_MOESM2_ESM.pdf]
